# Nucleus accumbens core chemogenetic excitation in male mice and chemogenetic inhibition in female mice reduced ethanol reward

**DOI:** 10.1186/s13293-025-00745-0

**Published:** 2025-08-28

**Authors:** Amy E. Chan, Gillian S. Driscoll, Zaynah Usmani, Angela R. Ozburn

**Affiliations:** 1 Department of Behavioral Neuroscience, Oregon Health & Science University, Portland Alcohol Research Center, Portland, OR 97239 USA; 2 Veterans Affairs Portland Health Care System, Research and Development Service, Portland, OR 97239 USA

**Keywords:** DREADDs, Nucleus accumbens, Ethanol reward, Conditioned place preference, Intoxication

## Abstract

**Background:**

Women tend to progress from initial alcohol use to dependence more rapidly than men, a phenomenon known as the “telescoping effect”. This suggests different consequences of early alcohol use, which can impact the development of an Alcohol Use Disorder (AUD). Previous evidence demonstrated that nucleus accumbens core (NAcC) chemogenetic manipulations resulted in opposite effects on binge-like drinking [stimulation decreased ethanol intake in C57BL/6J (B6) females, while inhibition decreased intake in males]. In humans, ethanol cue conditioning is linked to the positive subjective effects of alcohol intake and intoxication. We tested the hypothesis that chemogenetic manipulation of NAcC activity alters ethanol reward (measured by conditioned place preference, CPP) in a sex-specific manner.

**Methods:**

In Experiment 1, surgery naïve B6 mice (*n* = 11–12/sex/treatment) underwent an ethanol CPP protocol and were administered the *D*esigner *R*eceptors *E*xclusively *A*ctivated by *D*esigner *D*rugs (DREADD) actuator clozapine-N-oxide (CNO, 1 mg/kg) or vehicle prior to ethanol (2 g/kg) conditioning. In Experiment 2, B6 mice underwent surgery to deliver control (mCherry), excitatory (hM3Dq), or inhibitory (hM4Di) DREADDs to the NAcC (*n* = 8–13/sex/treatment). After recovery, mice underwent ethanol CPP as in Experiment 1. CPP was conducted in a 3-chamber apparatus. Time spent in each chamber was recorded during the pre-test (before conditioning), and the test (after 4 ethanol and 4 saline conditioning sessions). Data were analyzed separately by sex, viral condition, and treatment with a 2-way RM ANOVA [factors: Time (repeated measure), Chamber].

**Results:**

Both surgery naïve (Experiment 1) and mCherry-expressing female and male B6 mice condition similarly to an intoxicating dose of ethanol and CNO did not interfere with ethanol CPP in the absence of DREADDs. Experiment 2 revealed that NAcC chemogenetic *stimulation* prevented ethanol CPP in males, while NAcC chemogenetic *inhibition* prevented ethanol CPP in females.

**Conclusions:**

NAcC chemogenetic manipulations alter ethanol reward differently in male and female B6 mice. Together with prior work, we demonstrate that NAcC activity has a sex-specific role during ethanol reward and consumption. Evidence of sex differences in ethanol reward may help future research to uncover the mechanisms underlying the “telescoping effect” and why women have an increased risk for developing an AUD.

**Supplementary Information:**

The online version contains supplementary material available at 10.1186/s13293-025-00745-0.

## Abstract (plain language summary)

Women tend to progress from initial alcohol use to alcohol addiction more rapidly than men, a phenomenon known as the “telescoping effect”. This suggests different consequences of early alcohol use in men and women, which can impact the development of an Alcohol Use Disorder (AUD). A better understanding of sex differences in brain functions will contribute to more effective AUD treatments for both women and men. Prior research indicated that a brain region, the nucleus accumbens core (NAcC), is important for alcohol drinking and the rewarding feelings experienced with alcohol drinking. In mouse models of alcohol drinking, changing NAcC activity impacts drinking differently in females and males (*increased* activity reduced drinking in females, *decreased* activity reduced drinking in males). We hypothesized that NAcC activity influenced how much alcohol mice drank because it affects the rewarding effects of alcohol. This study measured how changing NAcC activity alters alcohol reward in female and male mice. Critical control experiments demonstrated that female and male mice find an intoxicating dose of alcohol similarly rewarding, indicating that further results are not due to baseline sex differences in alcohol reward. We discovered that *increasing* NAcC activity reduced alcohol reward in male mice, while *decreasing* NAcC activity reduced alcohol reward in female mice. These results indicate that a brain region important for alcohol reward and drinking works differently in females and males. Evidence of sex differences in ethanol reward may help future research to uncover the mechanisms underlying the “telescoping effect”.

**Supplementary Information:**

The online version contains supplementary material available at 10.1186/s13293-025-00745-0.

## Highlights

Nucleus accumbens core chemogenetic *stimulation* reduced ethanol reward in *male* mice.

Nucleus accumbens core chemogenetic *inhibition* reduced ethanol reward in *female* mice.

Male and female C57BL/6J mice condition similarly and reliably to an intoxicating dose of ethanol.

**Supplementary Information:**

The online version contains supplementary material available at 10.1186/s13293-025-00745-0.

## Background

Alcohol use and Alcohol Use Disorder (AUD) diagnoses have historically been more prevalent in men than women, though recently this gender gap has drastically narrowed [1–3]. Women tend to progress from initial alcohol use to AUD more rapidly than men, a phenomenon known as the “telescoping effect” [4, 5]. This suggests different consequences of early alcohol use in men and women, which may impact subsequent AUD development. Research into potential sex differences is critical, as women have more severe health outcomes in response to alcohol, including increased risk of developing cancer and higher death rates [6–9].

Rodent models of alcohol reward can further our understanding of these sex differences. Conditioned place preference (CPP) is often used to study the rewarding effects of alcohol in mice [10]. This task repeatedly pairs a stimulus (e.g. ethanol) with a distinct context, commonly tactile and/or visual cues. Following repeated pairings, or conditioning sessions, preference is exhibited as increased time spent in the reward associated context, relative to the non-reward associated context. Cue conditioning to ethanol reinforcement is a translationally relevant behavior. Though rats commonly form a conditioned place aversion to ethanol, there is evidence that under certain conditions (e.g. specific ethanol doses, alcohol-preferring strains) rats exhibit a modest ethanol CPP, indicating this behavior can be seen in other rodent species [11–13]. In men and women social drinkers, alcohol conditioning increased attention to alcohol-paired cues and was associated with increased liking of the alcoholic beverage consumed during conditioning sessions [14].

The nucleus accumbens core (NAcC) is an important addiction related brain region that regulates the rewarding and interoceptive effects of ethanol [15]. One study reported that lesions to the NAcC prior to conditioning (but not after conditioning) prevent ethanol CPP in male DBA/2J mice [16]. Moreover, NAcC chemogenetic inhibition increased sensitivity to the interoceptive effects of ethanol in male rhesus macaques [17]. This indicates that, in males, reductions to NAcC activity can change the rewarding value of ethanol in opposing directions, depending on species and behavioral task.

Several studies demonstrated sex-specific responses to ethanol in the nucleus accumbens (NAc). Female and male rats and mice exhibit different monoamine responses to ethanol, though the specific differences depend on species and route of ethanol administration [e.g. per os, i.p., or intra-NAc [18–22]. Following limited access binge-like ethanol drinking, male and female C57BL/6J (B6) mice exhibited distinct transcriptional responses in the NAc [23]. Female B6 mice also had greater engagement of NAc inputs following binge-like ethanol drinking than males [24]. NAcC chemogenetic inhibition increased binge-like ethanol intake in B6 females, but decreased intake in B6 males, while stimulation had opposite effects [females decreased intake, males had no change or increased intake [25–27]. Further, NAc chemogenetic stimulation increased (and inhibition decreased) intermittent access two-bottle choice ethanol intake in male rats, demonstrating that NAc chemogenetic stimulation can increase ethanol drinking in males across species and drinking models [28]. This evidence supports that the male and female NAcC is differentially responsive to intoxicating amounts of ethanol.

Based on these findings, we hypothesized that manipulations to NAcC activity influence ethanol reward differently in female and male B6 mice. First, in Experiment 1, we conducted a critical control experiment to establish that surgery naïve female and male B6 mice condition similarly to 2 g/kg ethanol in our 3-chamber conditioning apparatus. In Experiment 2, mice underwent surgery to deliver excitatory DREADDs (*D*esigner *R*eceptors *E*xclusively *A*ctivated by *D*esigner *D*rugs, hM3Dq), inhibitory DREADDs (hM4Di), or control virus (mCherry) bilaterally to the NAcC. Given that the two primary NAcC neuron types [dopamine type-1 (D1) and − 2 (D2) medium spiny neurons (MSNs)] work in tandem during associative learning, and both drive aversion resistant ethanol intake, DREADDs were expressed under a pan-neuronal promoter (hSyn) to assess holistic, rather than cell-type specific, NAcC function during ethanol reward [29, 30]. We hypothesized that the chemogenetic manipulations that decreased binge-like ethanol intake worked by reducing the rewarding effects of ethanol. Therefore, we predicted that NAcC chemogenetic inhibition in males, and stimulation in females, would reduce expression of ethanol CPP.

## Materials & methods

### Mice

Adult female and male B6 (10 weeks old, 24.0 g ± 0.35 at start of experiment) mice were obtained from Jackson Laboratory (Sacramento, CA) and housed at 22 ± 1^°^C in EcoFresh paper bedding (JSR Pharma, Patterson, NY) on a 12 h/12 h reverse light/dark cycle with lights off at 0730 (PST). Mice were acclimated to this reverse light/dark cycle for 5–7 days prior to testing or surgery. Mice were group housed (2–4/cage) and had *ad libitum* access to tap water and standard chow (5LOD, PMI Nutrition International, St. Louis, MO). All protocols were approved by the Portland Veteran Affairs Medical Center Institutional Animal Care and Use Committee and conducted in accordance with the National Institutes of Health Guidelines for the Care and Use of Laboratory Animals.

### Drugs

Clozapine-n-oxide (CNO; 1 mg/kg, HelloBio, Princeton, NJ) was dissolved in sterile saline (0.9% NaCl, Baxter Laboratories, Deerfield, IL). This dose was chosen for its ability to activate DREADDs with few off-target effects [25]. Ethanol (Decon Labs, King of Prussia, PA) was diluted to 20% (v/v) in sterile saline. CNO and ethanol solutions were prepared fresh daily.

### Ethanol conditioned place preference

**Fig. 1:**
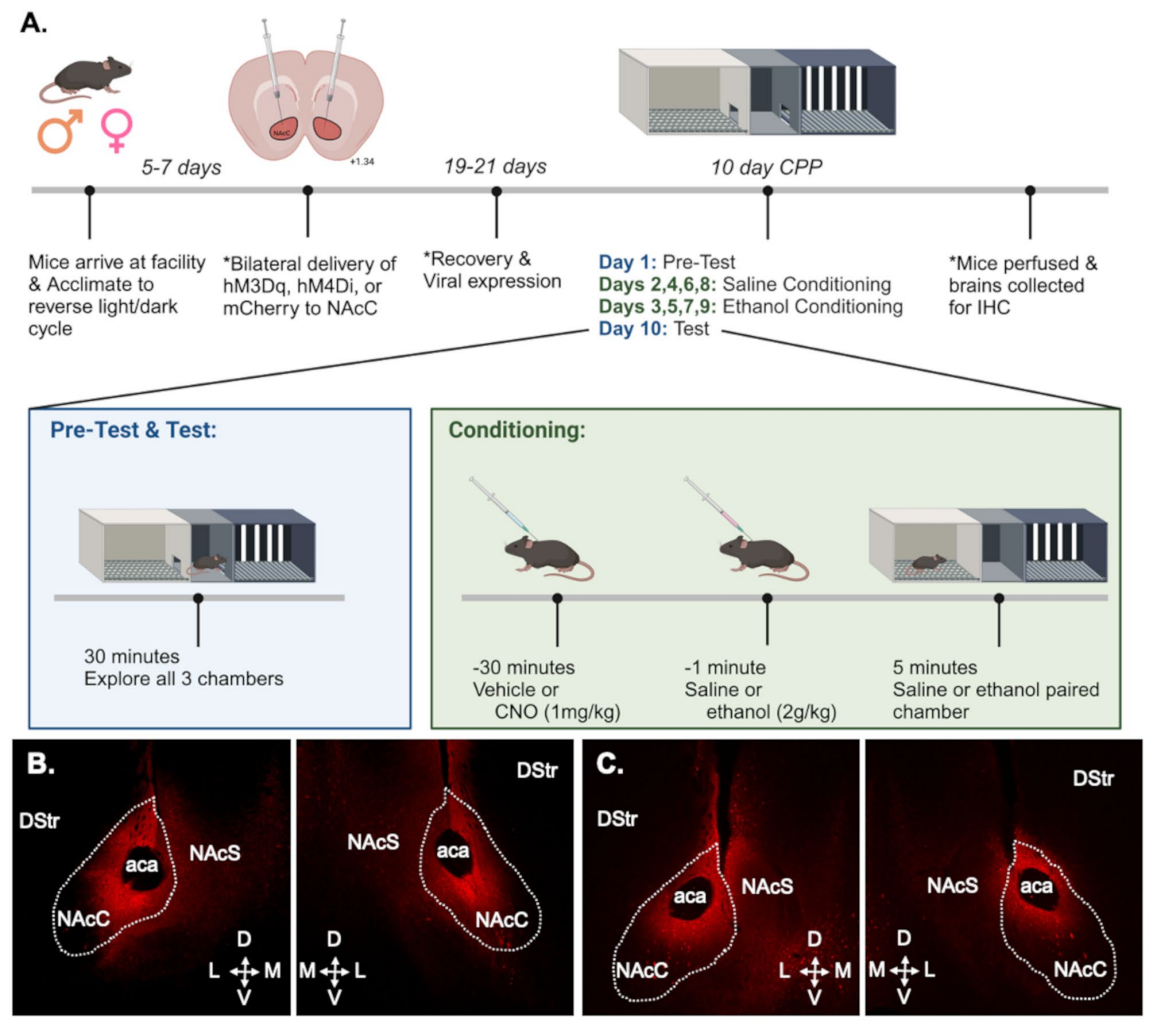
Experimental timeline and representative viral expression. **(a)** Timeline for Experiments 1 and 2. *Completed for Experiment 2 only (Created with Biorender.com). **(b)** Example viral expression from a male hM3Dq mouse. **(c)** Example viral expression from a male hM4Di mouse. Dotted lines denote approximate NAcC boundaries. aca, anterior commissure; D, dorsal; DStr, dorsal striatum; L, lateral; M, medial; NAcS, nucleus accumbens shell; V, ventral

All experiments were conducted starting 3 h into the dark cycle in 3-chamber MedAssociates CPP apparatus with adjustable doors separating the chambers (see Fig. 1A for experimental timeline). A 3-chamber apparatus allows mice to more actively choose between side chambers, unlike the binary choice available in a 2-chamber apparatus. Side chambers had distinct visual and tactile cues (white walls + grid floor or black and white striped walls + rod floor) and the middle chamber had gray walls + solid flooring. Compound cues (visual and tactile) accelerate CPP acquisition in B6 mice to levels similar to a mouse strain known for robust CPP [DBA/2J [31]. Time and locomotor activity in each chamber was recorded (via horizontally aligned infrared beams) for each session. On day 1 (pre-test), mice were placed in the middle chamber and allowed to freely explore all chambers for 30 min. After the pre-test, mice were pseudo-randomly assigned to a context for ethanol pairing in a counterbalanced and unbiased manner to ensure no baseline group differences in time spent on the ethanol paired side. Each mouse then underwent 8 conditioning sessions (4 ethanol, 4 saline), alternating daily, with CNO (1 mg/kg) or vehicle (saline) given 30 min prior to ethanol (2 g/kg), and vehicle given prior to saline. The 2 g/kg dose is an intoxicating dose of ethanol that was chosen because it results in similar blood ethanol concentrations as binge-like drinking [∼ 200 mg/dL [32, 33], and can produce ethanol CPP in several strains of mice [31, 34]. CNO was delivered prior to ethanol conditioning sessions to disrupt NAcC activity during ethanol intoxication, as NAcC activity is critical for acquisition of ethanol CPP and alters the interoceptive effects of ethanol [16, 17]. The order of conditioning sessions was not counterbalanced between mice (all mice received saline on sessions 1,3,5,7 and ethanol on sessions 2,4,6,8) in line with standard practice [10]. Ethanol rather than saline pairing the day prior to test may produce more robust CPP expression, though we did not address this possibility to reduce the potential for behavioral variability. On day 10 (test), mice were placed in the middle chamber with free access to both side chambers for 30 min.

### Experiment 1: Testing effect of CNO on acquisition of ethanol CPP in naïve female and male B6 mice

Female and male mice underwent ethanol CPP to determine whether (1) both sexes condition similarly to 2 g/kg ethanol, and (2) CNO alters acquisition of ethanol CPP. One mouse was excluded because of a baseline chamber preference > 75% (*n* = 11–12/sex/treatment).

### Experiment 2: Testing effects of NAcC chemogenetic manipulations on acquisition of ethanol CPP

Female and male mice received either excitatory (hM3Dq), inhibitory (hM4Di), or control (mCherry) virus to determine whether manipulating NAcC activity during acquisition alters ethanol CPP. Only mice with bilateral NAcC DREADD expression were included. Four mice expressing mCherry were excluded due to off target viral expression (*n* = 8–13/sex/virus/treatment).

### Stereotactic surgery

All mice in Experiment 2 underwent stereotactic surgery with isoflurane anesthesia (5% induction, 1–3% maintenance). Intracranial viral injections were carried out using standard procedures [25, 35, 36]. AAV2-hSyn-hM3D(Gq)-mCherry, AAV2-hSyn-hM4D(Gi)-mCherry, or AAV2-hSyn-mCherry (1.0µL/side, Addgene, Watertown MA), was injected bilaterally into the NAcC (10° angle, M/L ± 1.50, A/*P* + 1.50, D/V - 4.50 and − 4.00 mm from bregma) using a 33-gauge Hamilton syringe (Hamilton, Reno NV). Virus titers were 0.93-1.9 × 10^13^vg/mL. Virus was infused at a rate of 0.1µL/minute, and the needle was kept in place for an additional five minutes to allow for diffusion. 0.5µL of virus was delivered at -4.50 mm, and the remaining 0.5µL of virus was infused at -4.00 mm. For three days following surgery, mice were given ketofen (5 mg/kg, s.c.) and saline (0.5mL 0.9% s.c.) to aid in post-operative recovery. Experiments began 3 weeks after surgery to allow for viral expression (see Fig. 1A).

### Verification of viral expression

Following testing, all mice in Experiment 2 were deeply anesthetized with ketamine (200 mg/kg) and xylazine (20 mg/kg). Intracardial perfusions were performed with phosphate buffered saline (PBS) followed by 4% paraformaldehyde (PFA) in PBS. Brains were extracted and post-fixed in 4% PFA overnight before being submerged in cryoprotectant (30% glycerol in PBS + 0.02% sodium azide). Brains were sectioned at 30 μm on a freezing microtome (Leica, Wetzlar, Germany) and stored in PBS + 0.02% sodium azide. Immunohistochemistry was performed using rabbit anti-mCherry primary antibody (1:1000; 1 µg/mL; Abcam, Waltham, MA) and goat anti-rabbit IgG Alexa Fluor 594 secondary antibody (1:500; 4 µg/mL; ThermoFisher, Waltham, MA). Sections were counterstained and mounted using Vectashield with DAPI (Vector Labs, Burlingame, CA). Viral infusions were verified using a fluorescent microscope (Olympus BX60), as described in [25, 27, 35, 37, 38]. Immunohistochemistry was performed despite the viruses containing fluorescent tags to enable visualization of the full extent of viral spread without photobleaching of the endogenous fluorescent signal, similar to prior work [25, 27, 35, 37, 38]. See Figs. 1B&C for representative viral expression.

Image analysis was performed using ImageJ. For each section, an ROI was drawn around the maximal extent of viral expression and area was measured. In Microsoft Excel, area under the curve (AUC) was calculated for each hemisphere and summed to quantify total viral expression for each mouse. Since prior work indicated that extent of DREADD viral spread (within the NAcC and adjacent NAc shell and dorsal striatum) negatively correlated with ethanol discrimination [17], we quantified total viral expression in each mouse to determine whether differences in viral expression may contribute to differences in ethanol CPP.

### Data analysis

Data are shown as mean ± SEM. All significant main effects and interactions are reported, with post-hoc testing using Fisher’s LSD or Tukey’s multiple comparisons test when appropriate. An α *≤* 0.05 was considered significant. Data analysis and graphs were generated using GraphPad Prism 10. Analysis of locomotor activity is included in the supplement.

Our primary hypothesis was to determine whether chemogenetic manipulation of NAcC activity during acquisition impacts ethanol reward in either sex. Therefore, we analyzed data for each sex and viral group separately (unless otherwise specified). To determine whether mice of each condition (sex, viral group, treatment) formed an ethanol CPP, time spent in each chamber (ethanol paired, unpaired neutral, saline paired) during the pre-test and test were analyzed with a 2-way RM ANOVA [factors: Time (repeated measure), Chamber]. Ethanol conditioning was evidenced by a Chamber x Time interaction, whereby mice spent more time in the ethanol paired chamber during the test than pre-test, and more time in the ethanol than saline paired chamber during the test.

Next, to determine if CNO impacted ethanol CPP, data for each sex and viral condition (surgery naïve, mCherry, hM3Dq, hM4Di) was analyzed. The mCherry group controlled for any effect of virus, surgery, and CNO on ethanol CPP but was not directly compared to the experimental groups. Rather, each experimental group was compared to its direct control (i.e. vehicle treated mice of the same sex and viral group). Comparison of DREADD + CNO groups to a collapsed vehicle group (across viral conditions) was considered as an alternative approach but was not completed because of the availability of direct controls. Relative time spent in the ethanol paired chamber (seconds in ethanol paired – seconds in saline paired chamber) during the pre-test and test was analyzed using a 2-way RM ANOVA [factors: Time (repeated measure), Treatment]. Positive values indicated more time spent on the ethanol than saline paired side, while negative values indicated more time spent on the saline than ethanol paired side. An ethanol CPP was evidenced by a main effect of Time, whereby relative time spent in the ethanol paired side was greater during the test than pre-test. An effect of Treatment or Treatment x Time interaction indicated that CNO impacted ethanol CPP in this condition.

CPP score (difference between seconds spent on the ethanol-saline paired side during the test and pre-test) was analyzed using a 3-way ANOVA (factors: Surgery, Sex, Treatment) to directly compare CPP magnitude between surgery naïve and control viral conditions. This analysis provided a measure of how consistent and reliable ethanol CPP was in B6 mice across our experiments, and whether magnitude of CPP was comparable between sexes. Positive values indicated that relative time spent on the ethanol paired side increased between pre-test and test, while negative values indicated a reduction in relative time spent on the ethanol paired side following conditioning.

Mixed-effects ANOVA was used to assess potential differences in viral spread between treatment and hemisphere within each sex and viral condition [factors: Section (repeated measure), Treatment and Hemisphere]. A simple linear regression was used to determine whether CPP score correlated with total viral expression (AUC). For detailed discussion of these results, see Supplemental Results and Figures S3, 6, & 8.

## Results

### Experiment 1: Testing effect of CNO on acquisition of ethanol CPP in naïve female and male B6 mice

#### CNO did not alter acquisition of ethanol CPP in surgery naïve mice

**Fig. 2:**
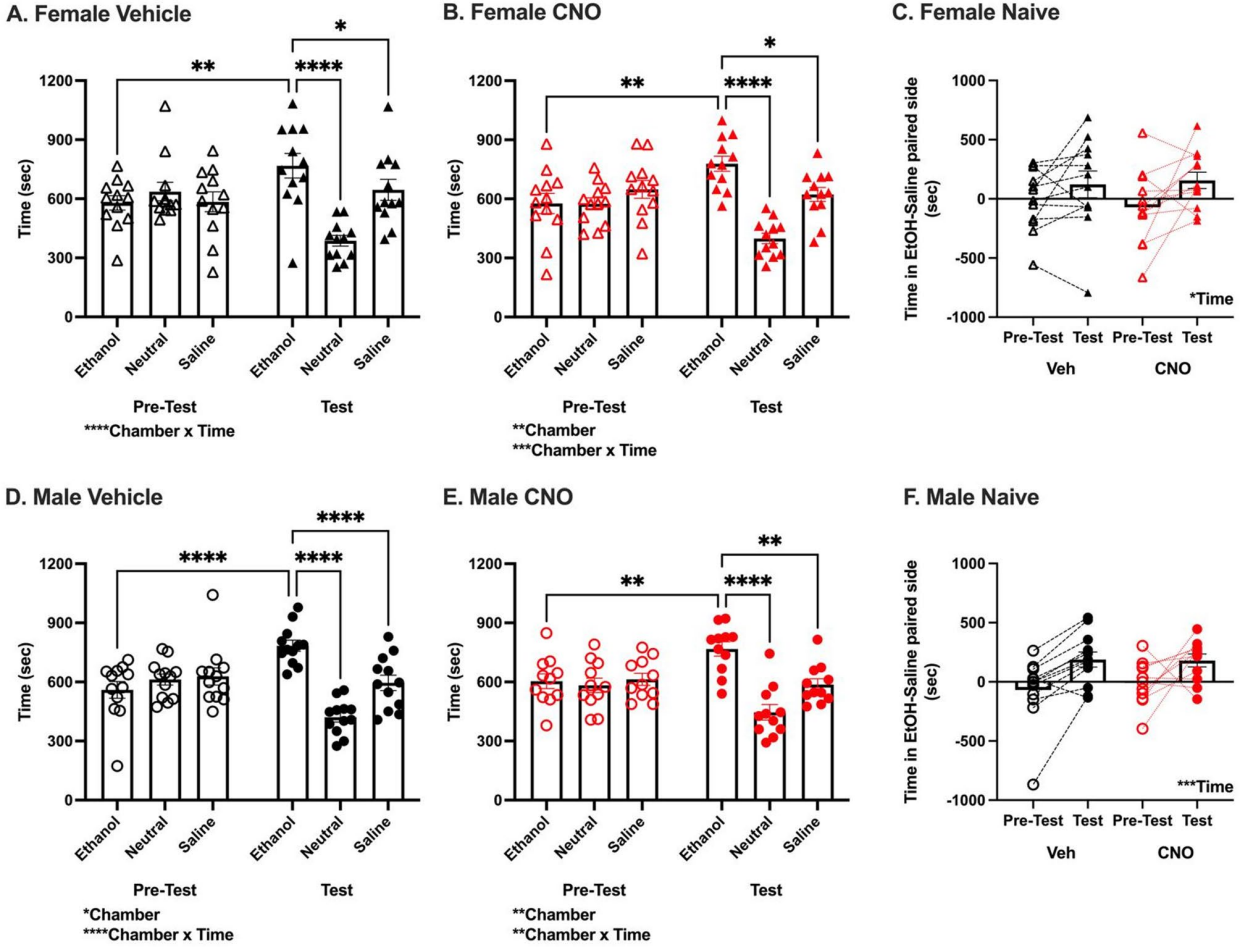
Surgery naïve females and males condition to 2 g/kg ethanol. **(a, b, d, e)** Time (sec) in each chamber (ethanol paired, unpaired neutral, saline paired) during pre-test and test. **(a)** Female Vehicle: Significant Chamber x Time interaction [*n* = 12, F_(2,22)_ = 16.01, ****p < 0.0001]. Selected significant pairwise comparisons shown. Significant pairwise comparisons not shown: effect of Time on time spent in neutral chamber (*p* < 0.001), test effect of Chamber on time spent in each chamber (saline > neutral, *p* < 0.001). **(b)** Female CNO: Significant main effect of Chamber [*n* = 12, F_(2,22)_ = 7.29, **p < 0.01], and Chamber x Time interaction [F_(2,22)_ = 10.15, ***p < 0.001]. Selected significant pairwise comparisons shown. Significant pairwise comparisons not shown: effect of Time on time spent in neutral chamber (*p* < 0.01), test effect of Chamber on time spent in each chamber (saline > neutral, *p* < 0.01). **(d)** Male Vehicle: Significant main effect of Chamber [*n* = 12, F_(2,22)_ = 3.97, *p < 0.05], and Chamber x Time interaction [F_(2,14)_ = 34.05, ****p < 0.0001]. Selected significant pairwise comparisons shown. Significant pairwise comparisons not shown: effect of Time on time spent in neutral chamber (*p* < 0.0001), test effect of Chamber on time spent in each chamber (saline > neutral, *p* < 0.0001). **(e)** Male CNO: Significant main effect of Chamber [*n* = 11, F_(2,20)_ = 6.23, **p < 0.01], and Chamber x Time interaction [F_(2,20)_ = 8.58, **p < 0.01]. Selected significant pairwise comparisons shown. Significant pairwise comparisons not shown: effect of Time on time spent in neutral chamber (*p* < 0.05), test effect of Chamber on time spent in each chamber (saline > neutral, *p* < 0.05). **(c, f)** Difference in time in ethanol and saline paired side (sec) during pre-test and test. **(c)** Female: Significant main effect of Time [F_(1,22)_ = 6.54, *p < 0.05], no effect of Treatment [F_(1,22)_ = 0.02, *p* = 0.88] or Time x Treatment interaction [F_(1,22)_ = 0.52, *p* = 0.48]. **(f)** Male: Significant main effect of Time [F_(1,21)_ = 16.49, ***p < 0.001], no effect of Treatment [F_(1,21)_ = 0.12, *p* = 0.74] or Time x Treatment interaction [F_(1,21)_ = 0.37, *p* = 0.55]. Open symbols denote pre-test, closed symbols denote test; females are denoted as triangles, males as circles; black symbols denote vehicle treated, red symbols denote CNO treated mice

First, we determined whether CNO impacted expression of ethanol CPP in either sex. In surgery naïve female vehicle treated mice, analysis of time spent in each chamber during pre-test and test revealed a significant Chamber x Time interaction [F_(2,22)_ = 16.01, *p* < 0.0001, Fig. 2A]. Post-hoc comparisons revealed mice spent significantly more time in the ethanol chamber during the test than pre-test (*p* < 0.01), and more time in the ethanol than saline and neutral chambers during the test (*p* < 0.05 and *p* < 0.0001, respectively). Similarly, in CNO treated females, analysis revealed a significant effect of Chamber [F_(2,22)_ = 7.29, *p* < 0.01] and Chamber x Time interaction [F_(2,22)_ = 10.15, *p* < 0.001, Fig. 2B]. Post-hoc analysis revealed mice spent significantly more time in the ethanol chamber during the test than pre-test (*p* < 0.01), and more time in the ethanol than saline and neutral chambers during the test (*p* < 0.05 and *p* < 0.0001, respectively). Across all surgery naïve females, analysis of relative time spent on the ethanol side during test versus pre-test revealed a significant effect of Time [F_(1,22)_ = 6.54, *p* < 0.05, Fig. 2C], but no effect of Treatment [F_(1,22)_ = 0.02, *p* = 0.88] or Time x Treatment interaction [F_(1,22)_ = 0.52, *p* = 0.48].

Likewise, in both vehicle and CNO treated surgery naïve males, analysis of time spent in each chamber during pre-test and test revealed a significant effect of Chamber [Vehicle: F_(2,22)_ = 3.97, *p* < 0.05; CNO: F_(2,20)_ = 6.23, *p* < 0.01] and Chamber x Time interaction [Vehicle: F_(2,14)_ = 34.05, *p* < 0.0001, Fig. 2D; CNO: F_(2,20)_ = 8.58, *p* < 0.01, Fig. 2E]. Post-hoc tests revealed mice spent significantly more time in the ethanol chamber during the test than pre-test (p’s < 0.01), and more time in the ethanol than saline and neutral chambers during the test (p’s < 0.01). Across all surgery naïve male mice, analysis of relative time spent on the ethanol side during test versus pre-test revealed a significant effect of Time [F_(1,21)_ = 16.49, *p* < 0.001, Fig. 2F] and no effect of Treatment [F_(1,21)_ = 0.12, *p* = 0.74] or Treatment x Time interaction [F_(1,21)_ = 0.37, *p* = 0.55]. These analyses indicated that both vehicle and CNO treated surgery naïve female and male mice formed and expressed an ethanol CPP and find an intoxicating (2 g/kg) dose of ethanol rewarding. Further, 1 mg/kg CNO did not significantly alter expression of ethanol CPP in either sex.

### Experiment 2: Testing effects of NAcC chemogenetic manipulations on acquisition of ethanol CPP

#### Females and males expressing control mCherry virus condition similarly to 2 g/kg ethanol, no effect of CNO

**Fig. 3:**
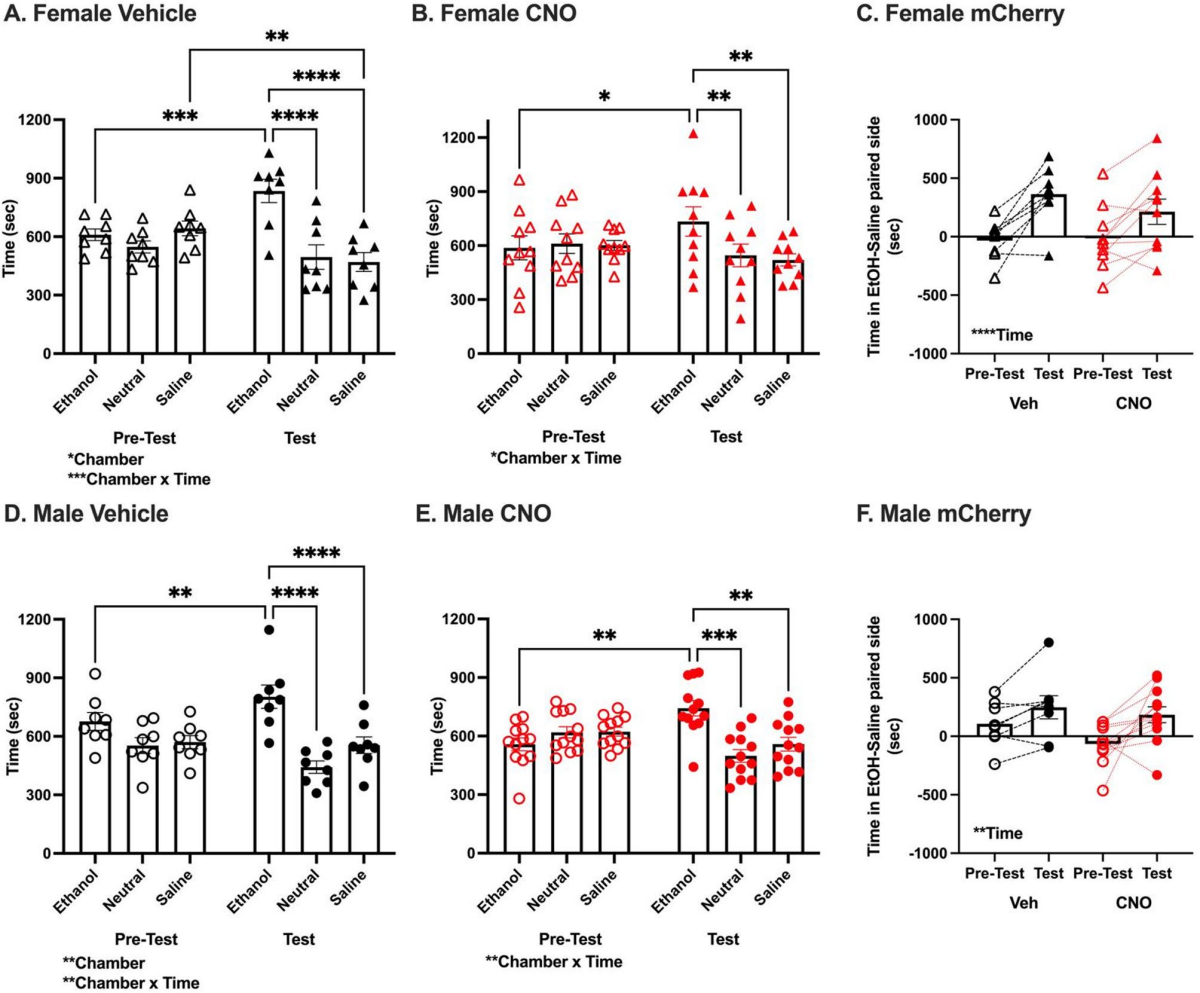
Females and males expressing control virus (mCherry) condition to 2 g/kg ethanol. **(a, b, d, e)** Time (sec) in each chamber (ethanol paired, unpaired neutral, saline paired) during pre-test and test. **(a)** Female Vehicle: Significant main effect of Chamber [*n* = 8, F_(2,14)_ = 4.47, *p < 0.05], and Chamber x Time interaction [F_(2,14)_ = 14.81, ***p < 0.001]. Significant pairwise comparisons shown. **(b)** Female CNO: Significant Chamber x Time interaction [*n* = 10, F_(2,18)_ = 4.36, *p < 0.05]. Significant pair-wise comparisons shown. **(d)** Male Vehicle: Significant main effect of Chamber [*n* = 8, F_(2,14)_ = 6.56, **p < 0.01], and Chamber x Time interaction [F_(2,14)_ = 8.78, **p < 0.01]. Selected significant pairwise comparisons shown. Significant pairwise comparisons not shown: effect of Time on time spent in neutral chamber (*p* < 0.05), pre-test effect of Chamber on time spent in each chamber (ethanol > neutral, *p* < 0.01; ethanol > saline, *p* < 0.05), test effect of Chamber on time spent in each chamber (saline > neutral, *p* < 0.05). **(e)** Male CNO: Significant Chamber x Time interaction [*n* = 12, F_(2,22)_ = 8.15, **p < 0.01]. Selected significant pairwise comparisons shown. Significant pairwise comparison not shown: effect of Time on time spent in neutral chamber (*p* < 0.05). **(c, f)** Difference in time in ethanol and saline paired side (sec) during pre-test and test. **(c)** Female: Significant main effect of Time [F_(1,16)_ = 29.86, ***p < 0.0001], no effect of Treatment [F_(1,16)_ = 0.33, *p* = 0.57] or Time x Treatment interaction [F_(1,16)_ = 2.21, *p* = 0.16]. **(f)** Male: Significant main effect of Time [F_(1,18)_ = 11.25, **p < 0.01], no effect of Treatment [F_(1,18)_ = 2.11, *p* = 0.16] or Time x Treatment interaction [F_(1,18)_ = 0.85, *p* = 0.37]. See Fig. 2 legend for symbol description

Next, we determined whether a history of surgery or NAcC viral expression impact the ability of female and male mice to form an ethanol CPP. In mCherry female vehicle mice, analysis of time spent in each chamber during pre-test and test revealed a significant effect of Chamber [F_(2,14)_ = 4.47, *p* < 0.05] and Chamber x Time interaction [F_(2,14)_ = 14.81, *p* < 0.001, Fig. 3A]. Post-hoc tests revealed mice spent significantly more time in the ethanol chamber during the test than pre-test (*p* < 0.001), more time in the ethanol than saline and neutral chambers during the test (p’s < 0.0001), and less time in the saline chamber during the test than pre-test (*p* < 0.01). Similarly, in CNO treated mCherry females, analysis revealed a significant Chamber x Time interaction [F_(2,18)_ = 4.36, *p* < 0.05, Fig. 3B]. Post-hoc comparisons indicated mice spent significantly more time in the ethanol chamber during the test than pre-test (*p* < 0.05), and more time in the ethanol than saline and neutral chambers during the test (p’s < 0.01).

In mCherry male vehicle mice, analysis of time spent in each chamber during pre-test and test revealed a significant effect of Chamber [F_(2,14)_ = 6.56, *p* < 0.01] and Chamber x Time interaction [F_(2,14)_ = 8.78, *p* < 0.01, Fig. 3D]. Post-hoc comparisons revealed mice spent significantly more time in the ethanol chamber during the test than pre-test (*p* < 0.01), and more time in the ethanol than saline and neutral chambers during the test (p’s < 0.0001). Likewise, in CNO treated mCherry males, analysis revealed a significant Chamber x Time interaction [F_(2,22)_ = 8.15, *p* < 0.01, Fig. 3E]. Post-hoc tests revealed mice spent significantly more time in the ethanol chamber during the test than pre-test (*p* < 0.01), and more time in the ethanol than saline and neutral chambers during the test (*p* < 0.01 and *p* < 0.001, respectively).

In both female and male mice expressing mCherry, analysis of relative time spent on the ethanol side during test versus pre-test revealed a significant effect of Time [Female: F_(1,16)_ = 29.86, *p* < 0.0001; Male: F_(1,18)_ = 11.25, *p* < 0.01; Fig. 3C&F]. There were no effects of Treatment [Female: F_(1,16)_ = 0.33, *p* = 0.57; Male: F_(1,18)_ = 2.11, *p* = 0.16] or Time x Treatment interactions [Female: F_(1,16)_ = 2.21, *p* = 0.16; Male: F_(1,18)_ = 0.85, *p* = 0.37] in either sex. Viral expression did not differ between groups and did not correlate with CPP score (Figure S3). This indicated that both vehicle and CNO treated females and males with a history of surgery formed and expressed an ethanol CPP.

#### Viral Surgery did not alter ethanol reward measured by CPP

To further validate that ethanol CPP is a reliable measure of ethanol reward in B6 mice, we compared CPP scores between surgery naïve and mCherry expressing female and male mice to determine whether CPP was similar between sexes and between these separate experiments. A 3-way ANOVA revealed no effects of Surgery [F_(1,77)_ = 0.74, *p* = 0.39], Sex [F_(1,77)_ = 0.33, *p* = 0.57], or Treatment [F_(1,77)_ = 0.01, *p* = 0.91], and no Surgery x Sex [F_(1,77)_ = 1.70, *p* = 0.20], Surgery x Treatment [F_(1,77)_ = 0.15, *p* = 0.70], Sex x Treatment [F_(1,77)_ = 0.20, *p* = 0.66], or Surgery x Sex x Treatment interactions [F_(1,77)_ = 3.21, *p* = 0.08; Figure S4]. Together, this indicated that B6 mice of both sexes reliably form an ethanol CPP of similar magnitude across experiments, with no effect of surgery or 1 mg/kg CNO treatment. These results further support that any sex differences in CPP following chemogenetic manipulations of NAcC activity are not due to baseline sex differences in ethanol reward, and that CNO alone did not interfere with acquisition of ethanol reward in this test.

#### NAcC chemogenetic stimulation reduced ethanol reward in males

**Fig. 4:**
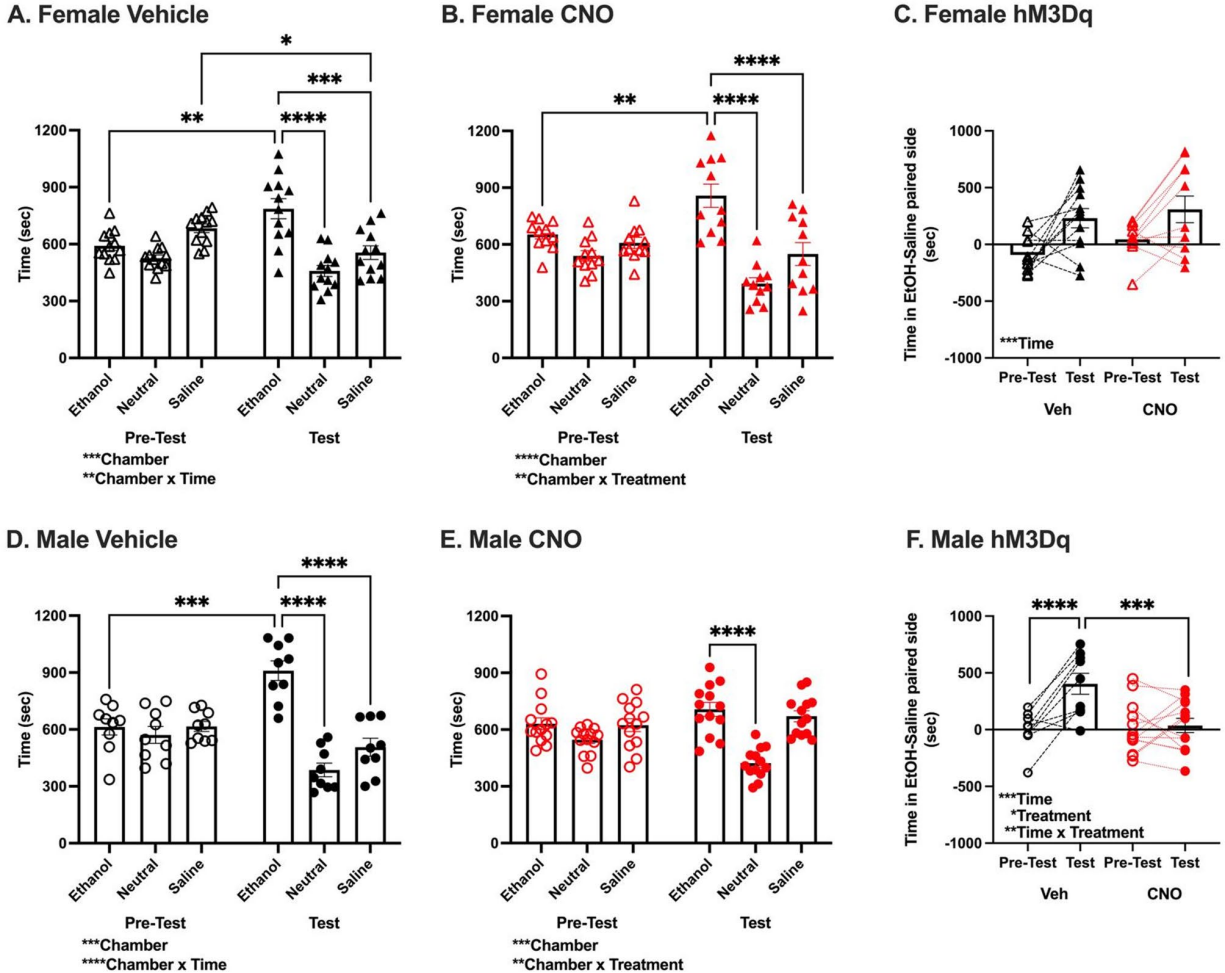
NAcC chemogenetic stimulation reduces ethanol CPP in males. **(a, b, d, e)** Time (sec) in each chamber (ethanol paired, unpaired neutral, saline paired) during pre-test and test. **(a)** Female Vehicle: Significant main effect of Chamber [*n* = 12, F_(2,22)_ = 12.37, ***p < 0.001], and Chamber x Time interaction [F_(2,22)_ = 9.49, **p < 0.01]. Selected significant pairwise comparisons shown. Significant pairwise comparison not shown: pre-test effect of Chamber on time spent in each chamber (saline > neutral *p* < 0.01). **(b)** Female CNO: Significant main effect of Chamber [*n* = 11, F_(2,20)_ = 11.21, ***p < 0.001], and Chamber x Time interaction [F_(2,20)_ = 11.10, ***p < 0.001]. Selected significant pairwise comparisons shown. Significant pairwise comparisons not shown: effect of Time on time spent in neutral chamber (*p* < 0.05), test effect of Chamber on time spent in each chamber (saline > neutral, *p* < 0.01). **(d)** Male Vehicle: Significant main effect of Chamber [*n* = 9, F_(2,16)_ = 11.69, ***p < 0.001], and Chamber x Time interaction [F_(2,16)_ = 19.19, ****p < 0.0001]. Selected significant pairwise comparisons shown. Significant pairwise comparison not shown: effect of Time on time spent in neutral chamber (*p* < 0.01). **(e)** Male CNO: Significant main effect of Chamber [*n* = 13, F_(2,24)_ = 10.63, ***p < 0.001], and Chamber x Time interaction [F_(2,24)_ = 7.82, **p < 0.01]. Selected significant pairwise comparisons shown. Significant pairwise comparisons not shown: effect of Time on time spent in neutral chamber (*p* < 0.01), pre-test effect of Chamber on time spent in each chamber (ethanol > neutral, *p* < 0.05), test effect of Chamber on time spent in each chamber (saline > neutral, *p* < 0.0001). **(c, f)** Difference in time in ethanol and saline paired side (sec) during pre-test and test. **(c)** Female: Significant main effect of Time [F_(1,21)_ = 15.75, ***p < 0.001], no effect of Treatment [F_(1,21)_ = 1.70, *p* = 0.21] or Time x Treatment interaction [F_(1,21)_ = 0.17, *p* = 0.69]. **(f)** Male: Significant main effect of Time [F_(1,20)_ = 15.94, *p* < 0.001], Treatment [F_(1,20)_ = 4.75, *p* < 0.05], and Time x Treatment interaction [F_(1,20)_ = 11.96, *p* < 0.01]. Pairwise comparisons shown (***p < 0.001, ****p < 0.0001). See Fig. 2 legend for symbol description

Since we established that both female and male B6 mice find an intoxicating dose of ethanol (2 g/kg) similarly rewarding, we next determined whether NAcC chemogenetic simulation alters ethanol reward in either sex. In vehicle and CNO treated female hM3Dq mice, analysis of time spent in each chamber during pre-test and test revealed a significant effect of Chamber [Vehicle: F_(2,22)_ = 12.37, *p* < 0.001; CNO: F_(2,20)_ = 11.21, *p* < 0.001] and Chamber x Time interaction [Vehicle: F_(2,22)_ = 9.49, *p* < 0.01, Fig. 4A; CNO: F_(2,20)_ = 11.10, *p* < 0.001, Fig. 4B]. Post-hoc analysis revealed mice spent significantly more time in the ethanol chamber during the test than pre-test (p’s < 0.01), and more time in the ethanol than saline and neutral chambers during the test (p’s < 0.001). In vehicle treated hM3Dq females, post-hoc tests also revealed mice spent significantly less time in the saline chamber during the test than pre-test (*p* < 0.05). Across all female hM3Dq mice, analysis of relative time spent on the ethanol side during test versus pre-test revealed a significant effect of Time [F_(1,21)_ = 15.75, *p* < 0.001; Fig. 4C], but no effect of Treatment [F_(1,21)_ = 1.70, *p* = 0.21] or Time x Treatment interaction [F_(1,21)_ = 0.17, *p* = 0.69]. This indicated that both vehicle and CNO treated females expressing hM3Dq formed and expressed an ethanol CPP.

In male hM3Dq vehicle mice, analysis of time spent in each chamber during pre-test and test revealed a significant effect of Chamber [F_(2,16)_ = 11.69, *p* < 0.001] and Chamber x Time interaction [F_(2,16)_ = 19.19, *p* < 0.0001, Fig. 4D]. Post-hoc comparisons revealed mice spent significantly more time in the ethanol chamber during the test than pre-test (*p* < 0.001), and more time in the ethanol than saline and neutral chambers during the test (p’s < 0.0001). In CNO treated hM3Dq males, analysis also revealed a significant effect of Chamber [F_(2,24)_ = 10.63, *p* < 0.001] and Chamber x Time interaction [F_(2,24)_ = 7.82, *p* < 0.01, Fig. 4E]. However, post-hoc tests indicated mice spent significantly more time in the ethanol than neutral chamber during the test (*p* < 0.0001), but no significant differences in time spent in the ethanol chamber during the pre-test versus test (*p* = 0.06), or between time spent in ethanol and saline chambers during the test (*p* = 0.35). Across all male hM3Dq mice, analysis of relative time spent on the ethanol side during test versus pre-test revealed significant effects of Time [F_(1,20)_ = 15.94, *p* < 0.001], Treatment [F_(1,20)_ = 4.74 *p* < 0.05], and Time x Treatment interaction [F_(1,20)_ = 11.96, *p* < 0.01, Fig. 4F]. Post-hoc tests revealed significant differences between pre-test and test in the vehicle group (*p* < 0.0001), and between vehicle and CNO group tests (*p* < 0.001). This demonstrated that vehicle treated males formed and expressed an ethanol CPP, while CNO treated males did not.

Viral expression did not differ between groups and did not correlate with CPP score (Figure S6). Together, this supports that NAcC chemogenetic stimulation reduced the rewarding effects of an intoxicating dose of ethanol (measured through reduced ethanol CPP) in males, but not females.

#### NAcC chemogenetic inhibition reduced ethanol reward in females

**Fig. 5:**
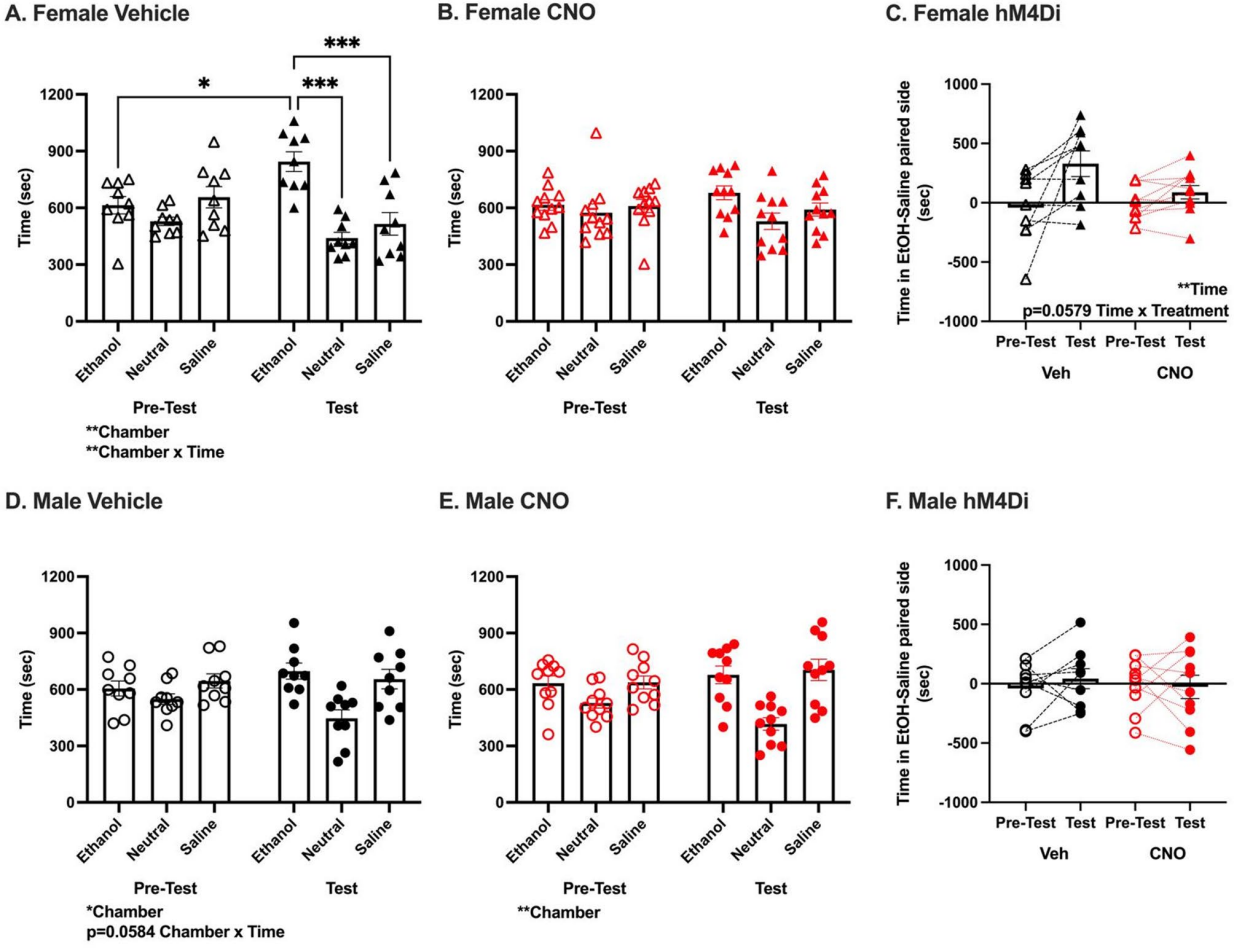
NAcC chemogenetic inhibition reduced ethanol CPP in females. **(a, b, d, e)** Time (sec) in each chamber (ethanol paired, unpaired neutral, saline paired) during pre-test and test. **(a)** Female Vehicle: Significant main effect of Chamber [*n* = 9, F_(2,16)_ = 9.19, **p < 0.01], and Chamber x Time interaction [F_(2,16)_ = 6.24, **p < 0.01]. Significant pairwise comparisons shown. **(b)** Female CNO: no significant main effects of Chamber [*n* = 11; F_(2,20)_ = 1.59, *p* = 0.22] or Chamber x Time interaction [F_(2,20)_ = 1.12, *p* = 0.35] **(d)** Male Vehicle: Significant main effect of Chamber [*n* = 9, F_(2,16)_ = 4.11, *p < 0.05], and modest Chamber x Time interaction [F_(2,16)_ = 3.41, *p* = 0.058]. **(e)** Male CNO: Significant main effect of Chamber [*n* = 10, F_(2,18)_ = 7.11, **p < 0.01], no significant Chamber x Time interaction [F_(2,18)_ = 3.096, *p* = 0.07]. **(c, f)** Difference in time in ethanol and saline paired side (sec) during pre-test and test. **(c)** Female: Significant main effect of Time [F_(1,18)_ = 10.15, **p < 0.01], modest Time x Treatment interaction [F_(1,18)_ = 4.10, *p* = 0.058]. **(f)** Male: No significant effects of Time [F_(1,17)_ = 0.19, *p* = 0.67], Treatment [F_(1,17)_ = 0.03, *p* = 0.86], or Time x Treatment interaction [F_(1,17)_ = 0.58, *p* = 0.46]. See Fig. 2 legend for symbol description

Lastly, we determined whether NAcC chemogenetic inhibition altered ethanol reward in female and male B6 mice. In female hM4Di vehicle mice, analysis of time spent in each chamber during pre-test and test revealed a significant effect of Chamber [F_(2,16)_ = 9.19, *p* < 0.01] and Chamber x Time interaction [F_(2,16)_ = 6.24, *p* < 0.01, Fig. 5A]. Post-hoc comparisons revealed mice spent significantly more time in the ethanol chamber during the test than pre-test (*p* < 0.05) and more time in the ethanol than saline and neutral chambers during the test (p’s < 0.001). In CNO treated hM4Di females, there was no significant effect of Chamber [F_(2,20)_ = 1.59, *p* = 0.22] or Chamber x Time interaction [F_(2,20)_ = 1.12, *p* = 0.35; Fig. 5B]. Across all female hM4Di mice, analysis of relative time spent on the ethanol side during test versus pre-test revealed a significant effect of Time [F_(1,18)_ = 10.15, *p* < 0.01, Fig. 5C], and modest Time x Treatment interaction [F_(1,18)_ = 4.10, *p* = 0.0579]. These analyses indicated that vehicle treated females expressing hM4Di formed and expressed an ethanol CPP, while CNO treated female mice did not.

In male hM4Di vehicle mice, analysis of time spent in each chamber during pre-test and test revealed a significant effect of Chamber [F_(2,16)_ = 4.11, *p* < 0.05] and modest Chamber x Time interaction [F_(2,16)_ = 3.41, *p* = 0.0584, Fig. 5D]. In male hM4Di CNO treated mice, there was a significant effect of Chamber [F_(2,18)_ = 7.11, *p* < 0.01, Fig. 5E] but no significant Chamber x Time interaction [F_(2,18)_ = 3.096, *p* = 0.07]. Across all male hM4Di mice, analysis of relative time spent on the ethanol side during test versus pre-test revealed no significant effects of Time [F_(1,17)_ = 0.19, *p* = 0.67], Treatment [F_(1,17)_ = 0.03, *p* = 0.86], or Time x Treatment interaction [F_(1,17)_ = 0.58, *p* = 0.46; Fig. 5F]. This indicated that neither vehicle nor CNO treated males expressing hM4Di formed an ethanol CPP.

Viral expression did not differ between groups and did not correlate with CPP score (Figure S8). These data demonstrated that NAcC chemogenetic inhibition reduced the rewarding effects of an intoxicating dose of ethanol (measured through reduced ethanol CPP) in females. Lack of conditioning in the male hM4Di vehicle group prevents us from concluding that NAcC chemogenetic inhibition also disrupted ethanol reward in males.

## Discussion

To understand whether the NAcC has a sex-specific role in ethanol reward, we chemogenetically manipulated NAcC activity during acquisition and measured the impact of these manipulations on ethanol CPP expression. Our results found that NAcC chemogenetic manipulations altered the rewarding effects of ethanol differently in females and males. In females, NAcC chemogenetic inhibition reduced ethanol CPP, while in males stimulation reduced ethanol CPP. These results are not attributable to baseline sex-differences in ethanol reward or effects of CNO, as both surgery naïve and control virus expressing mice condition similarly.

Present results contradict our initial hypothesis and support the alternate hypothesis that the previously described NAcC chemogenetic manipulations increased binge-like ethanol intake by reducing ethanol reward [25–28]. NAcC chemogenetic stimulation in males and inhibition in females potentially increased ethanol drinking to compensate for the reduced rewarding effects of ethanol. These results also indicated that reduced drinking in the females with NAcC chemogenetic stimulation was not through changes to ethanol reward. Frequently, female mice of different strains consume more ethanol than males during binge-like drinking tasks [for example: [39–43]. This suggests there could be differences in the underlying neurobiology of ethanol reward that produce different drinking patterns. Our results add to the literature on sex differences in ethanol drinking and related behaviors [recently reviewed in [44] and support that NAcC activity influences binge-like drinking and conditioned ethanol reward differently in females and males.

Though distinct behaviors and routes of ethanol administration (per os versus i.p) were used in ethanol intake vs. CPP experiments, several methodological considerations link these studies conceptually. First, the 2 g/kg ethanol dose results in intoxicating blood ethanol concentrations comparable to those achieved during a binge-like drinking session [∼ 200 mg/dL [32, 33]. Second, we conducted behavioral studies 3 h into the dark cycle, comparable to when mice had ethanol access during a Drinking-In-the-Dark procedure [45]. Finally, B6 mice were used, despite requiring more contextual cues than other strains (e.g. DBA/2J) to achieve comparable CPP expression [31, 46]. Our study utilized a CPP apparatus with distinct tactile and visual cues, which our lab and others have shown to reliably elicit CPP in B6 mice [31]. Across 15 inbred mouse strains, ethanol CPP was negatively correlated with sweetened ethanol consumption, in agreement with our results that found the chemogenetic manipulations that decreased ethanol reward also increased ethanol intake [47]. Altogether, we believe these experiments approximate some aspects of the neurobiological state of mice during binge-like drinking and offer compelling evidence that increased ethanol intake could in part be a consequence of a reduced ethanol reward.

Different consequences of NAcC chemogenetic manipulations on ethanol reward in females and males may suggest that different neurotransmitter systems or cell types are modulated by the DREADD manipulations. There is evidence that ethanol administration differentially affects monoamine levels in male and female mice, whereby 2 and 3 g/kg ethanol (i.p.) elicited increased NAc DOPAC (dopamine metabolite) and serotonin in male B6 mice, while these responses were blunted in females [22]. In contrast, following 3 g/kg ethanol, female B6 mice had higher NAc 5HTIAA (serotonin metabolite) levels than males [22]. Further, a 2 g/kg dose of ethanol increased tyrosine hydroxylase (necessary for dopamine synthesis) expression in female but not male B6 mice [21]. Together, DREADD manipulations may compound the differential effects ethanol has on neurotransmitter release and receptor expression to alter ethanol reward differently in females and males.

A wealth of literature supports that several neurotransmitter systems are involved in ethanol CPP. Antagonism of dopamine type-1 (D1) but not type-2 (D2) receptors systemically or intra-NAc reduced acquisition of ethanol CPP [34, 48, 49]. D1 antagonism prevented activation of adenyl cyclase and an influx of intracellular calcium, reducing neuronal activity [50]. NAcC NMDA receptor antagonism, which also prevents an influx of calcium, prevented ethanol CPP [51, 52]. All of these manipulations, which reduce NAc activity, were conducted in male mice. Our lack of conditioning in male hM4Di mice prevents us from concluding whether chemogenetic inhibition of NAcC activity with muscarinic DREADDs also reduces ethanol reward in males. However, our results that NAcC chemogenetic inhibition reduced ethanol reward in females could indicate that some of these neurotransmitter systems may also be relevant for ethanol reward in females.

Overexpression of Homer, a protein that traffics glutamate receptors to the cell membrane, increased ethanol CPP in male B6 mice [53]. The NAc oxytocin system also regulates ethanol CPP, as the analogue carbocetin or oxytocin receptor 1 overexpression reduced ethanol CPP [54]. Both manipulations increased NAcC activity but have opposing effects on ethanol reward, potentially through differences in magnitude of NAcC activity augmentation. Chemogenetic stimulation, which in the present study prevented ethanol CPP in males, has been reported to increase NAcC neuron firing rate by ∼ 30% [25]. In males, small increases in activity, like chemogenetic stimulation or oxytocin system manipulations, could prevent CPP, while larger increases in activity, like through increased glutamate signaling, could enhance ethanol reward. NAcC chemogenetic stimulation did not alter ethanol CPP in females, potentially indicating a celling effect and increased sensitivity to ethanol reward in females [supported by [18, 19, 55].

The pan-neuronal promoter, hSyn, used in this study means DREADDs can be expressed in all NAcC neuron types (D1 MSNs, D2 MSNs, cholinergic interneurons). Low striatal expression of G-protein inwardly rectifying potassium (GIRK) channels suggests that DREADDs primarily function through downstream effects of the Gα subunit [56]. Therefore, DREADDs likely augment or oppose the effects of a myriad of neurotransmitters important for ethanol CPP, particularly those signaling through G protein-coupled receptors. However, despite low levels of expression, activation of NAc hM4Di receptors with clozapine reduced neuronal firing rates in a GIRK dependent manner in male mice [57]. Together, this suggests that differences in how ethanol CPP is altered by hM4Di activation in males and females could be attributable to differences in GIRK expression or activity.

DREADDs are a powerful tool used to manipulate neuronal activity, but the effects of these manipulations on *in vivo* brain network connectivity are incompletely understood. Prior work in rodents and non-human primates (NHPs) confirmed in *ex vivo* slices that activation of hM4Di reduced [17] and hM3Dq increased NAc MSNs firing rate [25]. Further, in male B6 mice, prefrontal cortex chemogenetic inhibition reduced firing rate and increased resting state connectivity with other cortical and thalamic regions [58]. However, in male NHPs, amygdala chemogenetic inhibition with deschloroclozapine (DCZ), but not CNO, increased firing rates [59], and CNO activation of hM4Di receptors in the amygdala increased resting-state functional connectivity [60]. These studies indicate DREADD effects may be species, brain region, and actuator dependent, and may imply that our present study manipulates more than just NAcC activity. Though the present study and prior work demonstrate that NAcC activity is necessary for ethanol CPP [16], there are other reward-related brain regions anatomically connected to the NAcC that are important for conditioned ethanol reward [e.g. VTA: [61]; BNST: [62, 63]; amygdala: [51]; cortex: [64]; and midbrain [65]. Future work could determine if network activity compensates for disrupted NAcC activity and how this complements or conflicts with effects of NAcC activity on ethanol reward.

As suggested above, consideration of DREADD actuator and dose is important when interpreting results of chemogenetic manipulation studies. In naïve and mCherry mice, 1 mg/kg CNO did not alter acquisition of ethanol CPP, so results in Experiment 2 are most likely due to the action of CNO on DREADD receptors. Previous studies using much higher doses (10-20 mg/kg) found no effect of CNO on ethanol CPP in male DBA/2J mice [62]. However, CNO can back metabolize to clozapine and have off-target effects on dopamine and serotonin receptors [66]. We chose a low dose of CNO with low potential for off-target effects to facilitate comparison of these results with our previous work. Future studies should consider DREADD actuators, like DCZ, with more selective DREADD receptor affinity to eliminate this small potential concern [67].

One puzzling result was a lack of significant conditioning in male hM4Di vehicle mice, which prevents us from concluding whether NAcC chemogenetic inhibition also reduced ethanol CPP in males. Analysis of individual data showed 55% of vehicle, but only 40% of CNO hM4Di males increased time spent in the ethanol paired side during the test, suggesting more mice conditioned in the vehicle than CNO group. We ruled out possible explanations like small sample size (*n* = 9, comparable to male hM3Dq vehicle), effect of surgery (male mCherry and hM3Dq vehicle mice conditioned, no effect of surgery on CPP score between surgery naïve and mCherry mice, Figure S4), data variability (comparable standard deviation as other conditions), virus batch (no cell death or lesions evident in histology, female hM4Di vehicle mice conditioned), and viral titer (similar high titer in all experiments). Further, Experiment 2 was conducted by the same experimenter over several cohorts which contained mice of each group (e.g. viral condition, sex, and treatment), thus ruling out possible spurious effects of experimenter or time of year. Our choice of 2 g/kg ethanol is supported by the literature, as higher doses would not necessarily produce a stronger CPP, and lower doses could fail to or produce a weaker CPP [47, 68]. Future dose response studies could determine whether another dose of ethanol elicits CPP in male mice expressing hM4Di, and whether NAcC chemogenetic inhibition alters this CPP, but that is beyond the scope of the present study. Therefore, the only difference between male hM4Di and other mice in the study was the gene being expressed. This may suggest that bilateral hM4Di expression may interfere with ethanol reward behavior in male B6 mice. Future studies could inhibit NAcC activity through a different method (e.g. KORD DREADDs, inhibitory opsins) to determine whether reduced NAcC activity changes ethanol reward in males [69, 70].

One potential explanation for behavioral differences between female and male mice is the effect of the estrous cycle, which was not monitored in this study. Circulating sex hormones can influence neurotransmitter systems relevant to reward. For example, estrogen can modulate glutamate, dopamine, and serotonin signaling [71], high circulating estrogen (during diestrus) increased stimulated ventral tegmental area (VTA) dopaminergic neuron activity in female mice [72], and 17 β-estradiol potentiated amphetamine stimulated VTA dopamine release in ovariectomized female rats [73]. However, several studies show little-to-no effect of circulating ovarian hormones on ethanol drinking in female mice and rats [74–76]. Further, a study in Swiss Webster mice using an intoxicating dose of ethanol (1.8 g/kg) found no effect of estrous cycle on ethanol CPP [77]. The sample size (*n* = 8–13/condition) of the present study likely captured females in all stages of estrous during conditioning and testing, so our results are not likely to be specific to any phase of the cycle.

Variability in testosterone and the effects of testosterone on reward could also drive the reported sex differences. In humans, elevated testosterone is associated with increased neural reactivity to reward cues [78] and increased risk taking [79]. Like ovarian hormones, testosterone levels are not stable, as levels vary between group housed male mice because of social hierarchy [80], and fluctuate on both daily (humans and rodents) and monthly cycles [humans [81–83]. Together, while effects of sex hormones may contribute to some behavioral differences, there is not support for hormones fully explaining the different effects of NAcC activity on ethanol reward in females and males.

### Future directions

The present work fills critical gaps in the literature by demonstrating that chemogenetic manipulations of NAcC activity have opposing effects on conditioned ethanol reward in females and males. However, further work is necessary to address alternate explanations for these findings. First, additional studies should test whether these results are specific to ethanol reward, or due to general effects of NAcC manipulations on non-drug reward or learning. It will also be important to determine whether NAcC chemogenetic manipulations, in addition to changing ethanol intake and reward, change motivation to consume ethanol or ethanol satiation (measured through operant behaviors). Finally, since the NAcC can regulate ethanol aversion [30, 84, 85], as well as reward, reduced CPP could indicate that ethanol is either less rewarding, or more aversive. Given known sex differences in aversion resistant drinking [females consume more quinine adulterated ethanol than males [40, 86] it will be necessary to test whether modulation of NAcC activity also changes aversion (e.g. Conditioned Place Aversion) in a sex-specific manner.

## Conclusions

NAcC chemogenetic manipulations alter ethanol reward differently in male and female B6 mice. Present work demonstrates that the NAcC has a sex-specific role during ethanol reward and consumption. This evidence helps elucidate potential mechanisms of the “telescoping effect” and the increased risk for developing an AUD in women. Future studies could determine whether NAcC activity manipulation alter motivation for ethanol seeking, or if these results generalize to genetic models of risk for harmful drinking [e.g. High Drinking-in-the-Dark mice [87–89].

## Supplementary Information

Below is the link to the electronic supplementary material.

Supplementary Material 1

Supplementary Material 2

Supplementary Material 3

## Data Availability

All data generated or analyzed in the main figures of this study are included in this published article (Supplementary Tables 1&2). Data for supplementary analyses are available from the corresponding author upon reasonable request.
