# Supplementary material for: Nucleus accumbens core chemogenetic excitation in male mice and chemogenetic inhibition in female mice reduced ethanol reward
Source: Biol Sex Differ. 2025 Aug 28;16:66. doi: 10.1186/s13293-025-00745-0 (PMC12392586; doi:10.1186/s13293-025-00745-0)
Supplement: Supplementary file 3 — Supplementary Material 3 [file 13293_2025_745_MOESM3_ESM.pdf]

Supplemental Information

Nucleus accumbens core chemogenetic excitation in males and chemogenetic inhibition in females reduced ethanol reward

Chan *et al*.

Supplemental Materials & Methods:

Locomotor Activity Analysis

Locomotor activity during ethanol conditioning was analyzed separately for each sex and viral group (surgery naïve, mCherry, hM3Dq, hM4Di). Locomotor activity was recorded (via infrared beams) during conditioning and test sessions. To determine whether CNO affected locomotor activity, a 2-way repeated measures ANOVA (factors: Treatment, Session as repeated measure) was used to analyze locomotor activity during ethanol conditioning.

To determine whether differences in ethanol CPP between sexes could be attributable to differences in locomotor activity, a 2-way ANOVA (factors: Sex, Treatment) was used analyze total test session locomotor activity.

Supplemental Results:

Experiment 1:

CNO did not alter locomotor activity during ethanol conditioning

In surgery naïve female and male mice, analysis of locomotor activity during ethanol conditioning revealed no significant effects of Treatment [CNO or vehicle; Female: $F_{(1,22)}$=1.48, p=0.24; Male: $F_{(1,21)}$=3.27, p=0.08], conditioning Session [Female: $F_{(2.92,64.19)}$=2.31, p=0.09; Male: $F_{(2.84,59.71)}$=1.43, p=0.24], or Session x Treatment interaction [Female: $F_{(3,66)}$=0.29, p=0.83; Male: $F_{(3,63)}$=0.55, p=0.65; **Figure S1A&B**].

CNO treated surgery naïve mice had slightly lower locomotor activity during the test

Since prior work demonstrated that ethanol stimulated locomotor activity was negatively correlated with ethanol CPP (1), we examined whether there were differences in test locomotor activity. In surgery naïve mice, analysis of total test activity revealed a significant effect of Treatment [$F_{(1,43)}$=7.66, p<0.01], but no effect of Sex [$F_{(1,43)}$=0.01, p=0.94], or Sex x Treatment interaction [$F_{(1,43)}$=1.97, p=0.17], whereby CNO treated mice had slightly lower activity than vehicle treated controls (**Figure S1C**). These group differences were minor (Vehicle mean: 4210.2 ±83.5 SEM; CNO mean: 3908.1 ±71.5 SEM), and without effects of CNO on CPP Score or time spent on ethanol paired side likely not indicative of an effect of CNO on CPP expression.

Experiment 2:

Increased locomotor activity during conditioning in female mCherry mice

In female mCherry mice, 2-way RM ANOVA on locomotor activity during ethanol conditioning revealed an effect of Session [$F_{(2.096, 33.53)}=9.52$, $p<0.001$; **Figure S2A**], but no significant effect of Treatment [$F_{(1,16)}=0.56$, $p=0.46$] or Session x Treatment interaction [$F_{(3,48)}=1.66$, $p=0.19$]. Post hoc comparisons revealed higher locomotor activity during session 1 than 3 ($p<0.01$). In male mCherry mice, there were no effects of Session [$F_{(2.47,42.79)}=2.66$, $p=0.07$], Treatment [$F_{(1,18)}=0.12$, $p=0.74$], or Session x Treatment interaction [$F_{(3,52)}=9.52$, $p=0.43$; **Figure S2B**].

Test activity did not differ between female and male mCherry mice

Analysis of total test activity in vehicle and CNO treated female and male mCherry mice revealed no significant effect of Sex [$F_{(1, 34)}=0.00$, $p=0.99$], Treatment [$F_{(1, 34)}=0.55$, $p=0.46$] or Sex x Treatment interaction [$F_{(1,34)}=2.74$, $p=0.11$; **Figure S2C**].

mCherry viral spread did not differ between males and females, and did not correlate with CPP

In female mice expressing mCherry, there was no effect of Section [$F_{(1.67, 21.54)}=3.35$, $p=0.06$] or Treatment and Hemisphere [$F_{(3,23)}=0.78$, $p=0.52$] on viral spread (measured as area, mm^2). This indicated that viral expression throughout the NAcC (along the anterior-posterior axis) and extent of viral spread in each section was similar across both vehicle and CNO treated mice. Total NAcC viral expression did not significantly correlate with CPP Score in either vehicle [$R^2=0.07$, $F_{(1,6)}=0.47$, $p=0.52$] or CNO [$R^2=0.09$, $F_{(1,8)}=0.81$, $p=0.40$] treated mice.

In male mice expressing mCherry, there was a main effect of Section [$F_{(2.56, 42.66)}$=2.97, p<0.05] but no effect of Treatment and Hemisphere [$F_{(3,26)}$=0.33, p=0.80] on NAcC viral spread. This indicated that while there was variability in NAcC anterior-posterior viral spread, expression did not significantly differ between vehicle and CNO treated mice. Total NAcC viral expression did not significantly correlate with CPP Score in either vehicle [R^2=0.13, $F_{(1,6)}$=0.86, p=0.39] or CNO [R^2=0.06, $F_{(1,10)}$=0.62, p=0.45] treated mice.

Conditioning session and treatment impact locomotor activity in male hM3Dq mice

In female mice expressing hM3Dq in the NAcC, analysis of locomotor activity during ethanol conditioning revealed no effects of conditioning Session [$F_{(1.72, 35.01)}$=1.23, p=0.30], Treatment [$F_{(1, 21)}$=0.46, p=0.51], or Session x Treatment interaction [$F_{(3, 61)}$=0.56, p=0.30; **Figure S5A**].

In male hM3Dq mice, analysis of locomotor activity during ethanol conditioning revealed a significant effect of conditioning Session [$F_{(3,59)}$=10.63, p<0.0001], Treatment [$F_{(1,20)}$=4.92, p<0.05], and Session x Treatment interaction [$F_{(3,59)}$=3.053, p<0.05, **Figure S5B**]. Post-hoc tests indicate that vehicle group activity was higher during session 1 than 2, 3, or 4 (p's<0.01). CNO group activity during sessions 1 and 2 were higher than 4 (p's<0.05). Furthermore, the vehicle group had greater activity during session 1 than the CNO group (p<0.01).

Test activity did not differ between female and male hM3Dq mice

Analysis of total test activity in vehicle and CNO treated female and male hM3Dq mice revealed no significant effect of Sex [$F_{(1, 40)}$=0.25, p=0.62], Treatment [$F_{(1, 40)}$=0.36, p=0.55] or Sex x Treatment interaction [$F_{(1,40)}$=0.19, p=0.66; **Figure S5C**].

hM3Dq viral spread did not differ between males and females, and did not correlate with CPP

In female mice expressing hM3Dq, there was a significant main effect of Section [$F_{(9,240)}$=4.48, p<0.0001] but no effect of Treatment and Hemisphere [$F_{(3,42)}$=1.38, p=0.26] on NAcC viral spread. This indicates that while there was variability in viral spread throughout the anterior-posterior axis of the NAcC, expression did not significantly differ between vehicle and CNO treated mice. Total NAcC viral expression did not significantly correlate with CPP Score in vehicle [R^2=0.09, $F_{(1,10)}$=0.99, p=0.34] or CNO [R^2=0.01, $F_{(1,9)}$=0.13, p=0.73] treated mice.

In male mice expressing hM3Dq, there was a main effect of Section [$F_{(3.48,85.1)}$=3.95, p<0.01] but no effect of Treatment and Hemisphere [$F_{(3,40)}$=1.41, p=0.25] on viral spread in the NAcC. This indicates that despite variability in viral spread throughout the NAcC, expression did not differ between vehicle and CNO treated mice. Total NAcC viral expression did not significantly correlate with CPP Score in either vehicle [R^2=0.002, $F_{(1,7)}$=0.01, p=0.97] or CNO [R^2=0.28, $F_{(1,11)}$=0.28, p=0.61] treated mice.

Conditioning session and treatment impact locomotor activity in female hM4Di mice

In female mice expressing hM4Di in the NAcC, analysis of locomotor activity during conditioning sessions revealed significant effects of Session [$F_{(2.190,39.41)}$=5.580, p<0.01] and Treatment [$F_{(1,18)}$=6.46, p<0.05, **Figure S7A],** but no Session x Treatment interaction [$F_{(2.19,39.4)}$=1.71, p=0.19]. Post hoc tests indicate higher vehicle group activity during session 1 than 2 and 3 (p's<0.05). The vehicle group activity was higher than CNO group activity during sessions 1 and 2 (p's<0.05).

In male hM4Di mice, analysis of locomotor activity during conditioning sessions revealed no effects of conditioning Session [$F_{(2.172,36.2)}$=1.83, p=0.17], Treatment [$F_{(1,17)}$=2.61, p=0.12], or Session x Treatment interaction [$F_{(3,50)}$=2.49, p=0.07; **Figure S7B**].

Test activity did not differ between female and male hM4Di mice

Analysis of total test activity in vehicle and CNO treated female and male hM4Di mice revealed no significant effect of Sex [$F_{(1, 36)}$=2.27, p=0.14], Treatment [$F_{(1, 36)}$=0.39, p=0.54] or Sex x Treatment interaction [$F_{(1,36)}$=1.86, p=0.18; **Figure S7C**].

hM4Di viral spread did not differ between males and females, and did not correlate with CPP

In female mice expressing hM4Di there was a significant main effect of Section [$F_{(9,199)}$=7.97, p<0.0001] but no effect of Treatment and Hemisphere [$F_{(3,36)}$=0.34, p=0.80] on viral spread. This indicated that while there was variability in viral spread throughout the anterior-posterior axis of the NAcC, there was no significant difference in viral expression between vehicle and CNO treated mice. Total NAcC viral expression did

not significantly correlate with CPP Score in either vehicle [R^2=0.34, $F_{(1,7)}$=3.59, p=0.10] or CNO [R^2=0.30, $F_{(1,9)}$=3.76, p=0.08] treated mice.

In male mice expressing hM4Di, there was a main effect of Section [$F_{(9,177)}$=5.21, p<0.0001] but no effect of Treatment and Hemisphere [$F_{(3,34)}$=0.37, p=0.77] on NAcC viral spread. This indicated that while viral spread throughout the NAcC was variable, viral expression did not significantly differ between vehicle and CNO treated mice. Total NAcC viral expression did not significantly correlate with CPP Score in either vehicle [R^2=0.00, $F_{(1,7)}$=0.00, p=0.98] or CNO [R^2=0.08, $F_{(1,8)}$=0.65, p=0.44] treated mice.

Supplemental Figures:

Supplemental Figure 1. *Locomotor activity in surgery naïve mice.* (a,b) Locomotor activity during ethanol conditioning. (a) Female: No significant main effects [Treatment: $F_{(1,22)}=1.48$, $p=0.24$; Session: $F_{(2.92,64.19)}=2.31$, $p=0.09$; Session x Treatment interaction: $F_{(3,66)}=0.29$, $p=0.83$]. (b) Male: No significant main effects [Treatment: $F_{(1,21)}=3.27$, $p=0.08$; Session: $F_{(2.84,59.71)}=1.43$, $p=0.24$; Session x Treatment interaction: $F_{(3,63)}=0.55$, $p=0.65$]. (c) Total test activity. Main effect of Treatment [$F_{(1,43)}=7.66$, $p<0.01$], no effect of Sex [$F_{(1,43)}=0.01$, $p=0.94$], or Sex x Treatment interaction [$F_{(1,43)}=1.97$, $p=0.17$].

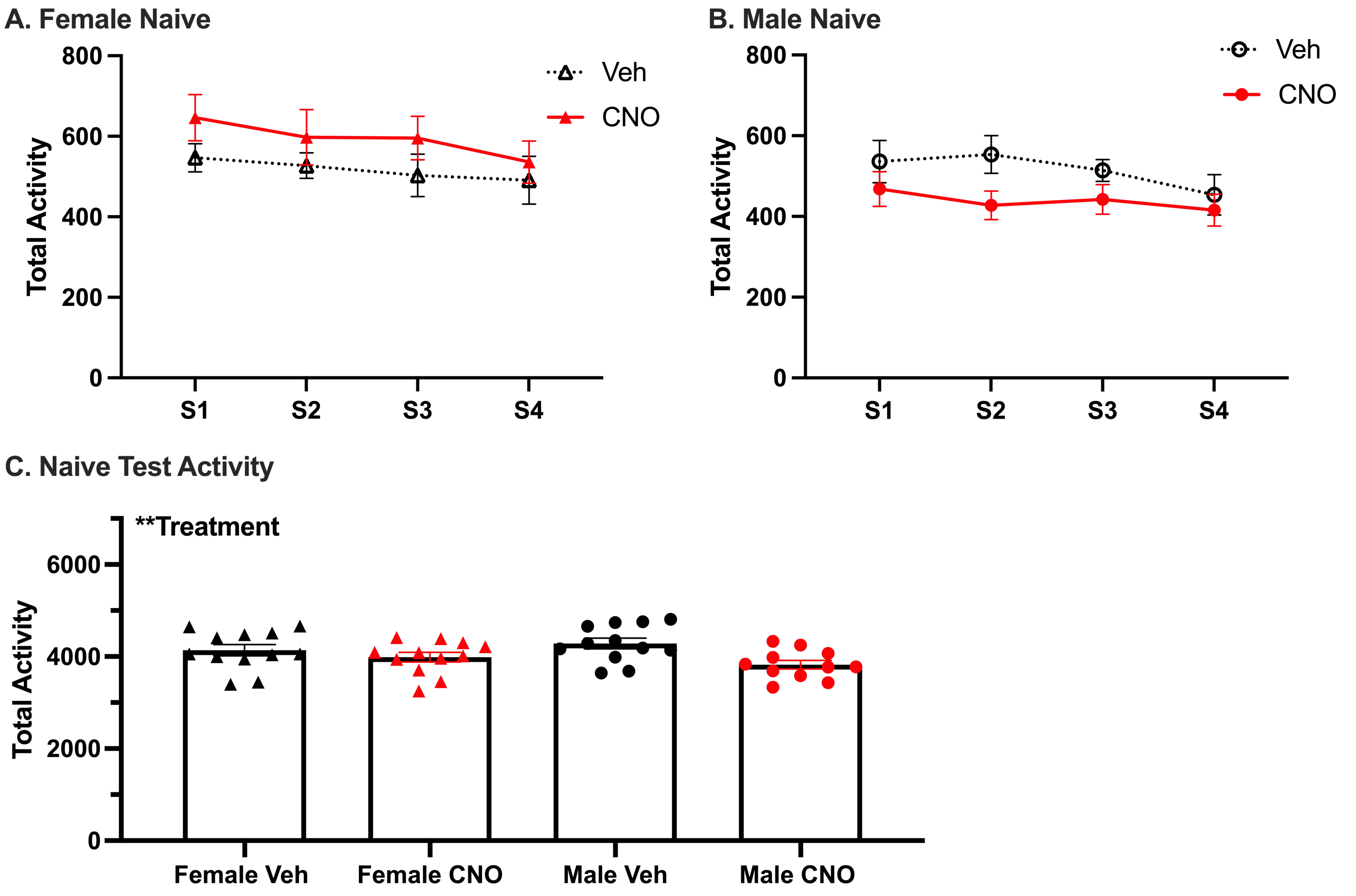

Supplemental Figure 1

Supplemental Figure 2. *Locomotor activity in mCherry mice.* (a,b) Locomotor activity during ethanol conditioning. (a) Female: Main effect of conditioning Session [$F_{(2.096, 33.53)}$=9.52, p<0.001], no effect of Treatment [$F_{(1,16)}$=0.56, p=0.46] or Session x Treatment interaction [$F_{(3,48)}$=1.66, p=0.19]. (b) Male: No significant effects of Session [$F_{(2.47,42.79)}$=2.66, p=0.07], Treatment [$F_{(1,18)}$=0.12, p=0.74], or Session x Treatment interaction [$F_{(3,52)}$=9.52, p=0.43]. (c) Total test activity. No significant effect of Sex [$F_{(1, 34)}$=0.00, p=0.99], Treatment [$F_{(1, 34)}$=0.55, p=0.46] or Sex x Treatment interaction [$F_{(1,34)}$=2.74, p=0.11].

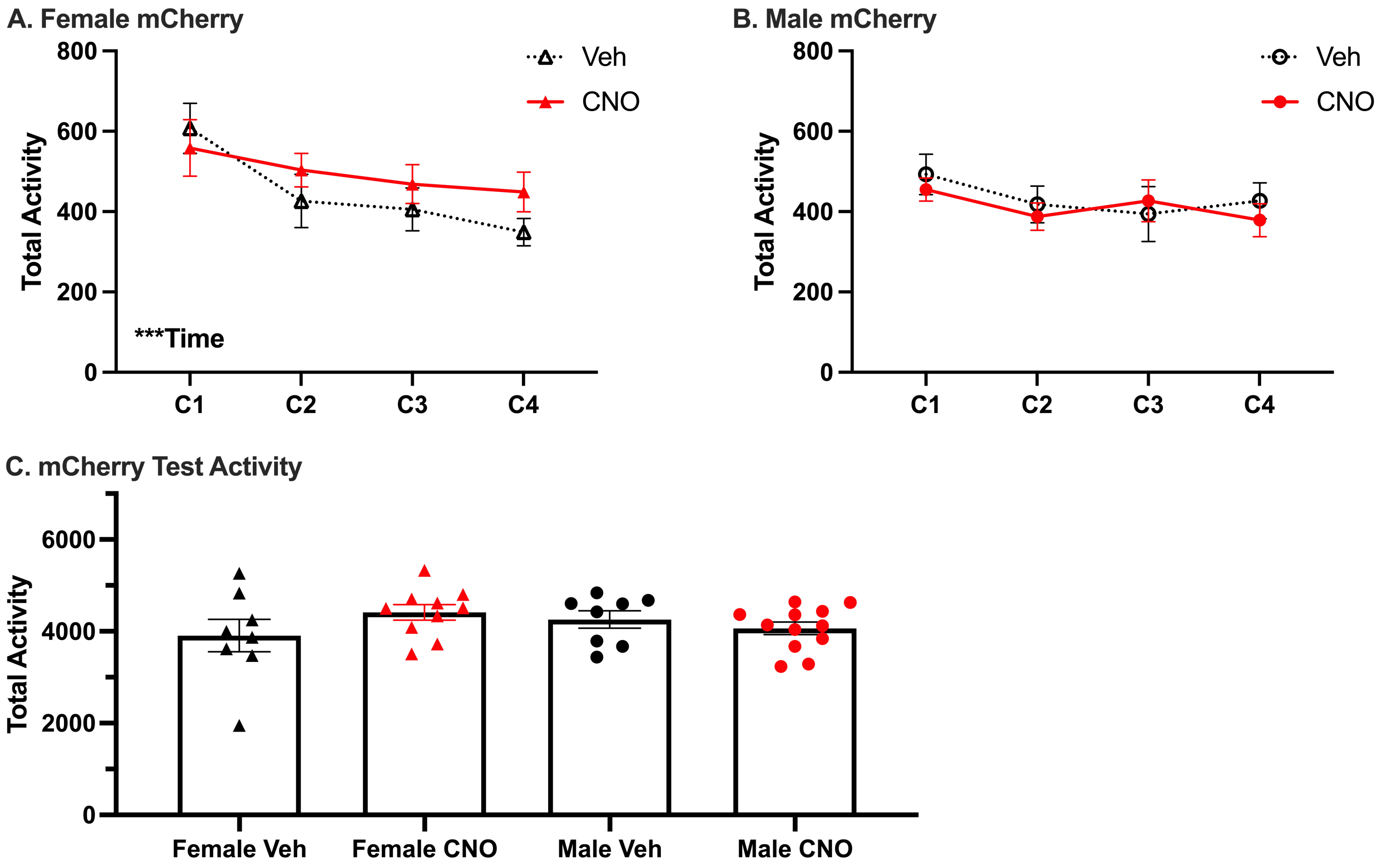

Supplemental Figure 2

Supplemental Figure 3. *Quantification of mCherry viral spread.* (a,b) Area of viral expression (mm^2) per Franklin & Paxinos atlas figure. (a) Female: no significant main effects of Section [$F_{(1.67, 21.54)}$=3.35, p=0.06] or Treatment and Hemisphere [$F_{(3,23)}$=0.78, p=0.52]. (b) Male: main effect of Section [$F_{(2.56,42.66)}$=2.97, $p<0.05$], no effect of Treatment and Hemisphere [$F_{(3,26)}$=0.33, p=0.80]. (c) Female: No significant correlations between CPP score and viral spread [Vehicle: R^2=0.07, $F_{(1,6)}$=0.47, p=0.52; CNO: R^2=0.09, $F_{(1,8)}$=0.81, p=0.40]. (d) Male: No significant correlations between CPP score and viral spread [Vehicle: R^2=0.13, $F_{(1,6)}$=0.86, p=0.39; CNO: R^2=0.06, $F_{(1,10)}$=0.62, p=0.45]. (e) Illustration of maximal mCherry viral expression in the NAcC. Images adapted from *The Mouse Brain in Stereotaxic Coordinates*, Franklin & Paxinos (2007).

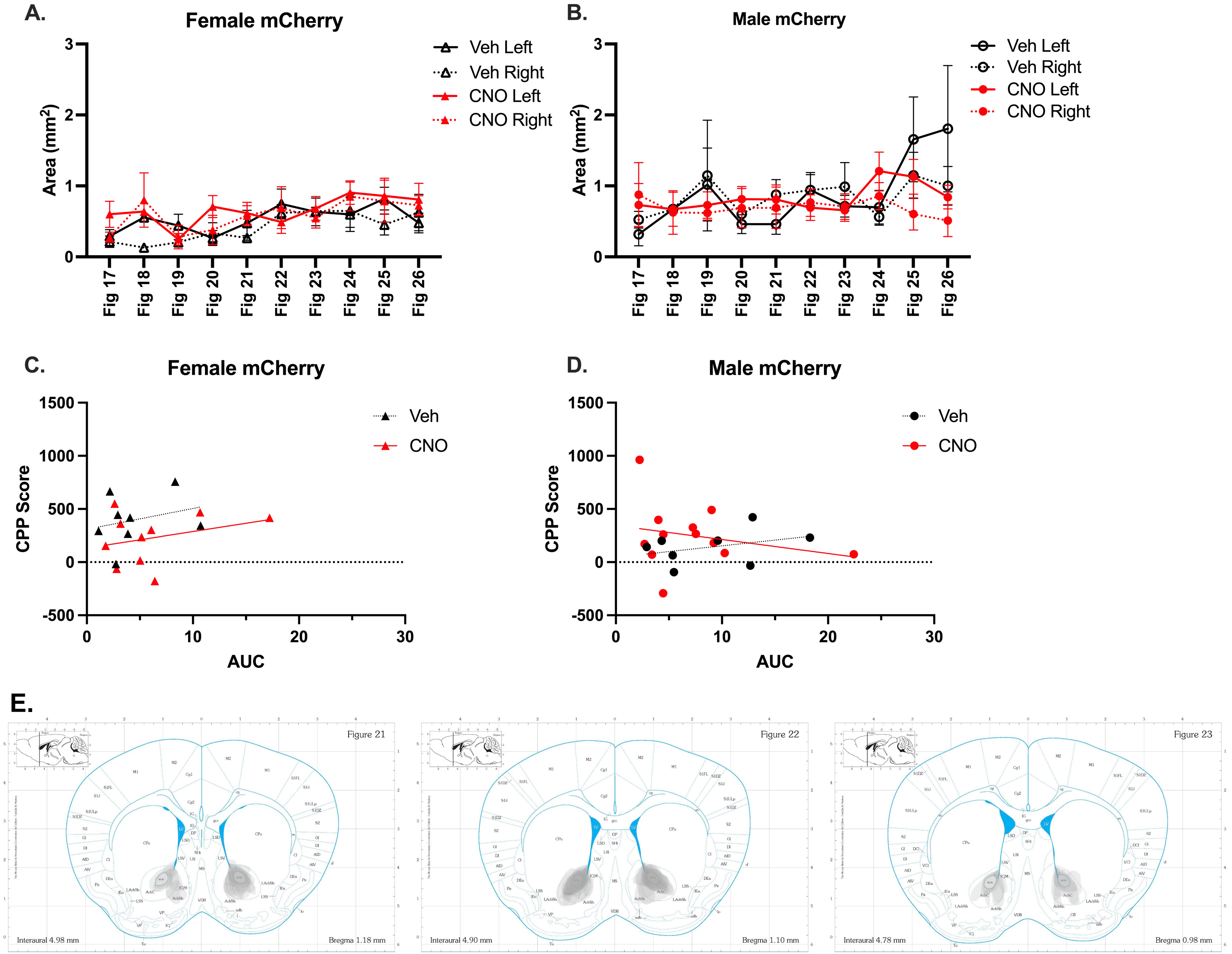

Supplemental Figure 3

Supplemental Figure 4. *Viral surgery did not alter ethanol CPP*. Comparison of CPP score in male and female mCherry (left) and surgery naïve mice (right). No significant effects of Surgery [$F_{(1,77)}$= 0.74, p=0.39], Sex [$F_{(1,77)}$= 0.33, p=0.57], or Treatment [$F_{(1,77)}$= 0.01, p=0.91], and no Surgery x Sex [$F_{(1,77)}$= 1.70, p=0.20], Surgery x Treatment [$F_{(1,77)}$= 0.15, p=0.70], Sex x Treatment [$F_{(1,77)}$= 0.20, p=0.66], or Surgery x Sex x Treatment interactions [$F_{(1,77)}$= 3.21, p=0.08].

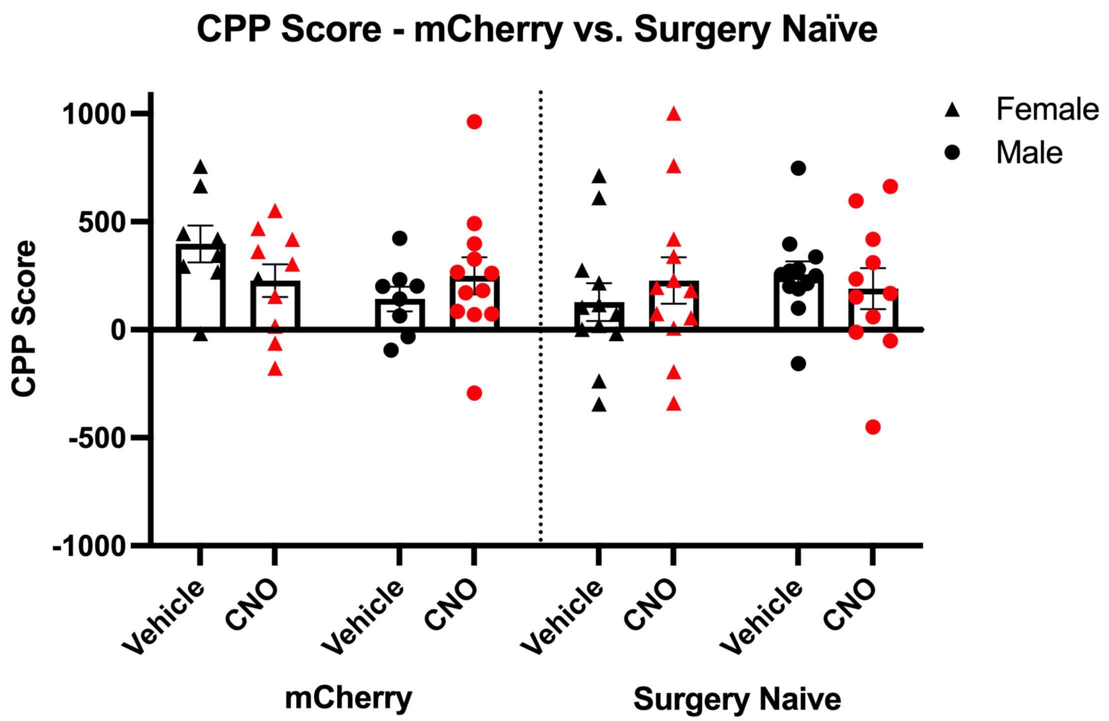

Supplemental Figure 4

Supplemental Figure 5. *Locomotor activity in hM3Dq mice.* (a,b) Locomotor activity during ethanol conditioning. (a) Female: No significant effects of Session [$F_{(1.72, 35.01)}$=1.23, p=0.30], Treatment [$F_{(1, 21)}$=0.46, p=0.51], or Session x Treatment interaction [$F_{(3, 61)}$=0.56, p=0.30]. (b) Male: Main effect of Treatment [$F_{(1,20)}$=4.92, *p<0.05], conditioning Session [$F_{(3,59)}$=10.63, p<0.0001], and Session x Treatment interaction [$F_{(3,59)}$=3.05, p<0.05]. (c) Total test activity. No significant effects of Sex [$F_{(1, 40)}$=0.25, p=0.62], Treatment [$F_{(1, 40)}$=0.36, p=0.55] or Sex x Treatment interaction [$F_{(1,40)}$=0.19, p=0.66].

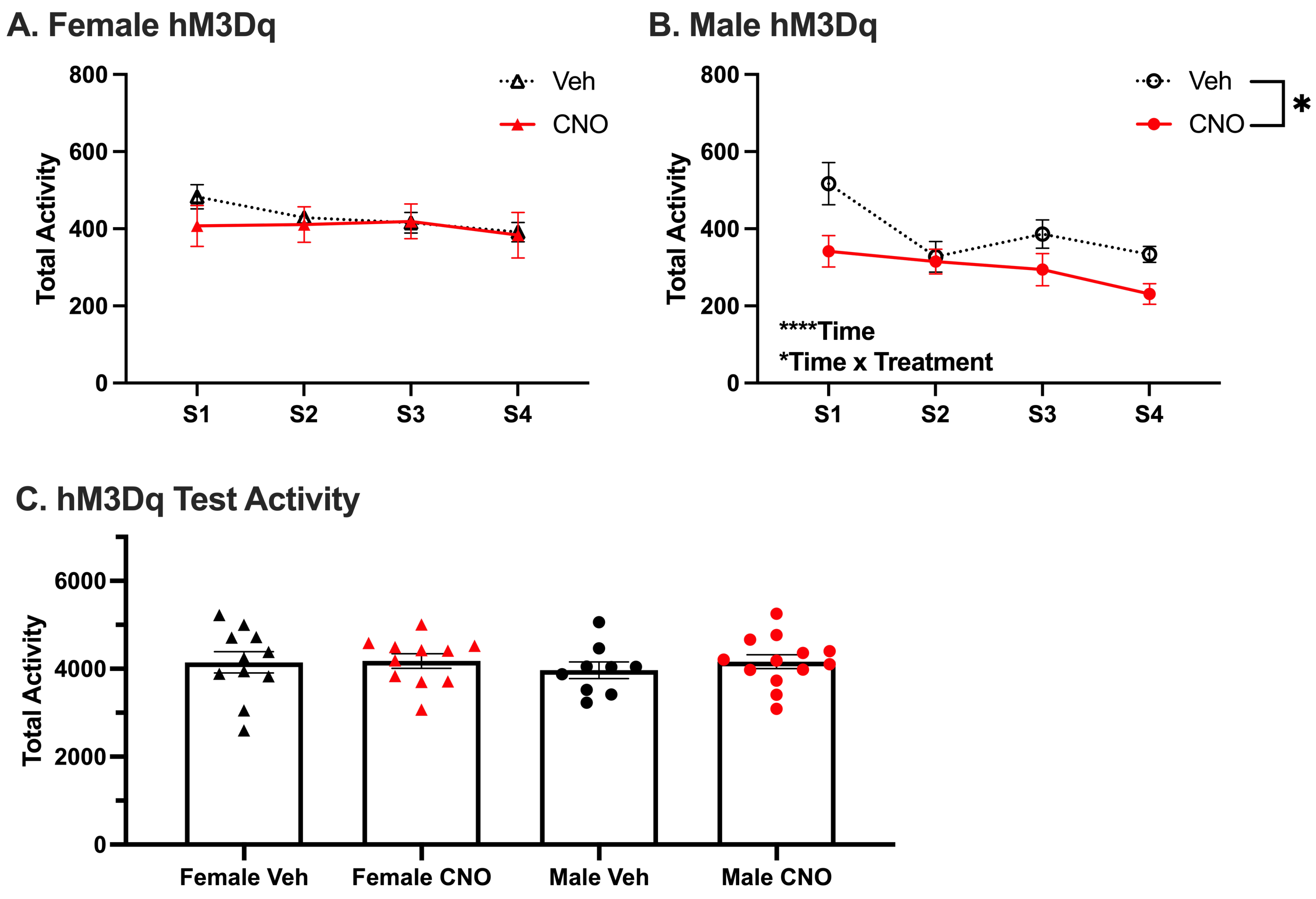

Supplemental Figure 5

Supplemental Figure 6. *Quantification of hM3Dq viral spread.* (a,b) Area of viral expression (mm^2) per Franklin & Paxinos atlas figure. (a) Female: main effect of Section [$F_{(9,240)}$=4.48, $p<0.0001$], no effect of Treatment and Hemisphere [$F_{(3,42)}$=1.38, p=0.26]. (b) Male: main effect of Section [$F_{(3.481,85.10)}$=3.95, $p<0.01$], no effect of Treatment and Hemisphere [$F_{(3,40)}$=1.41, p=0.25]. (c) Female: No significant correlations between CPP score and viral spread [Vehicle: R^2=0.09, $F_{(1,10)}$=0.99, p=0.34; CNO: R^2=0.01, $F_{(1,9)}$=0.13, p=0.73]. (d) Male: No significant correlations between CPP score and viral spread [Vehicle: R^2=0.002, $F_{(1,7)}$=0.01, p=0.97; CNO: R^2=0.28, $F_{(1,11)}$=0.28, p=0.61]. (e) Illustration of maximal hM3Dq viral expression in the NAcC. Images adapted from *The Mouse Brain in Stereotaxic Coordinates*, Franklin & Paxinos (2007).

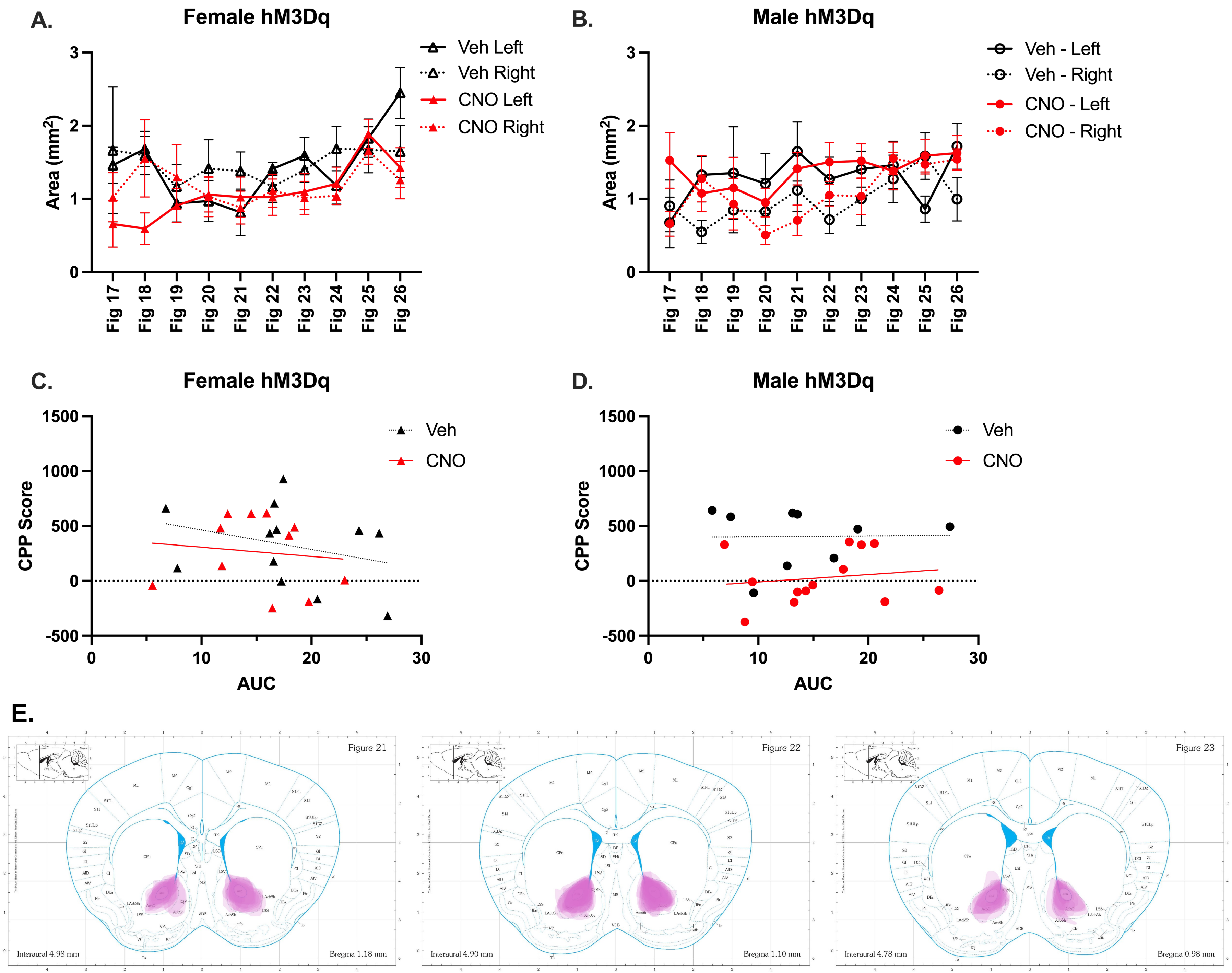

Supplemental Figure 6

Supplemental Figure 7. *Locomotor activity in hM4Di mice.* (a,b) Locomotor activity during ethanol conditioning. (a) Female: Main effect of Treatment [$F_{(1,18)}$=6.46, p<0.05] and conditioning Session [$F_{(2.190,39.41)}$=5.58, p<0.01]. (b) Male: No significant effects of Session [$F_{(2.172,36.2)}$=1.83, p=0.17], Treatment [$F_{(1,17)}$=2.61, p=0.12], or Session x Treatment interaction [$F_{(3,50)}$=2.49, p=0.07]. (c) Total test activity. No significant effects of Sex [$F_{(1, 36)}$=2.27, p=0.14], Treatment [$F_{(1, 36)}$=0.39, p=0.54], or Sex x Treatment interaction [$F_{(1,36)}$=1.86, p=0.18].

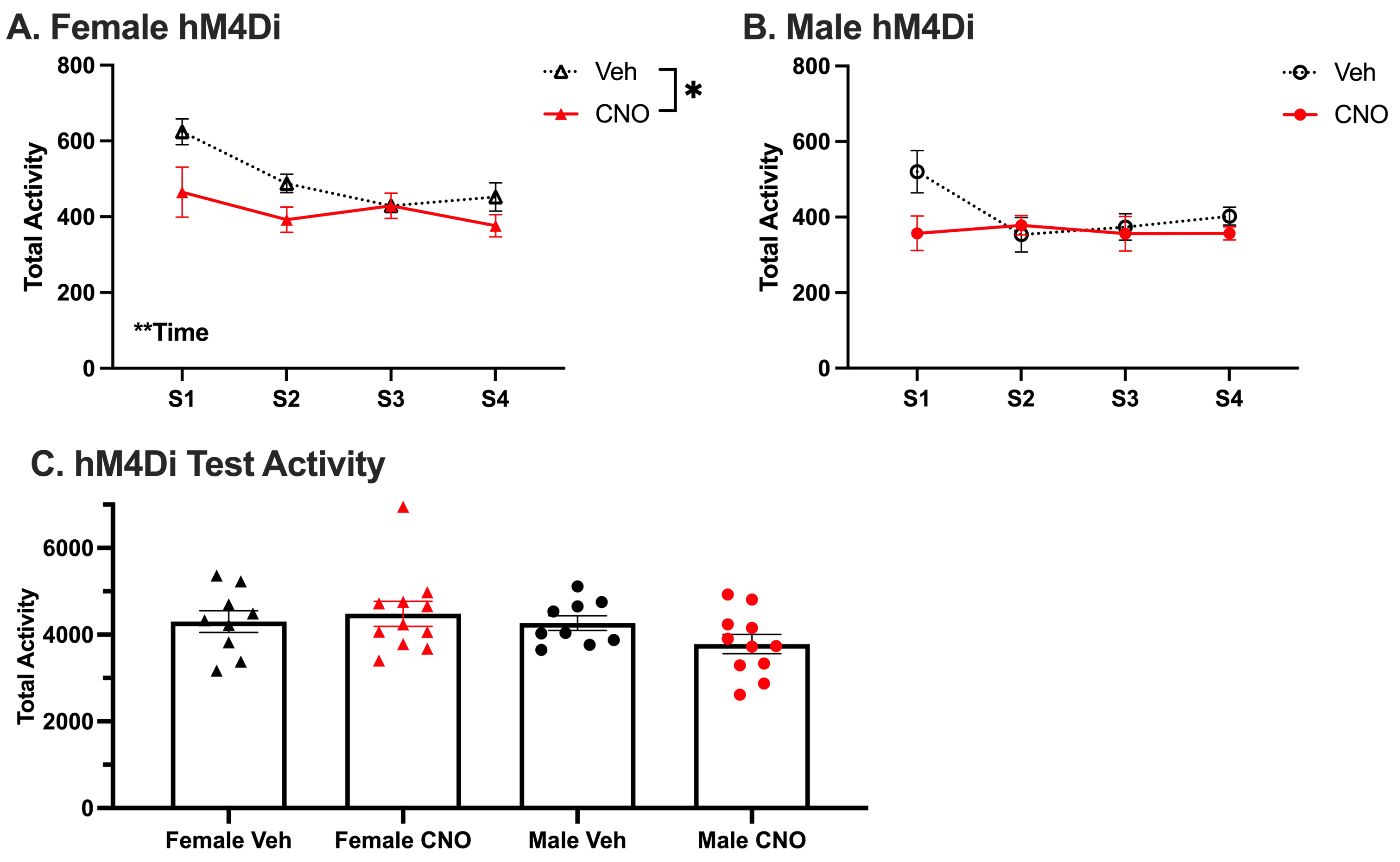

Supplemental Figure 7

Supplemental Figure 8. *Quantification of hM4Di viral spread.* (a,b) Area of viral expression (mm^2) per Franklin & Paxinos atlas figure. (a) Female: main effect of Section [$F_{(9,199)}$=7.97, p<0.0001], and Section x Treatment and Hemisphere interaction [$F_{(27,199)}$=1.62, p<0.05], but no effect of Treatment and Hemisphere [$F_{(3,36)}$=0.34, p=0.80]. Follow-up analysis indicated this significant interaction was driven by a Section x Hemisphere interaction in the CNO mice [$F_{(9,109)}$=2.87, p<0.01]. (b) Male: main effect of Section [$F_{(9,177)}$=5.21, p<0.0001], and Section x Treatment x Hemisphere interaction [$F_{(27,177)}$=1.63, p<0,05], but no effect of Treatment and Hemisphere [$F_{(3,34)}$=0.37, p=0.77]. Follow-up analysis indicated this significant interaction was driven by a Section x Hemisphere interaction in the CNO mice [$F_{(9,95)}$=2.86, p<0.01]. (c) Female: No significant correlations between CPP score and viral spread [Vehicle: R^2=0.34, $F_{(1,7)}$=3.59, p=0.10; CNO: R^2=0.30, $F_{(1,9)}$=3.76, p=0.08]. (d) Male: No significant correlations between CPP score and viral spread [Vehicle:R^2=0.00, $F_{(1,7)}$=0.00, p=0.98; CNO: R^2=0.08, $F_{(1,8)}$=0.65, p=0.44]. (e) Illustration of maximal hM4Di viral expression in the NAcC. Images adapted from *The Mouse Brain in Stereotaxic Coordinates*, Franklin & Paxinos (2007).

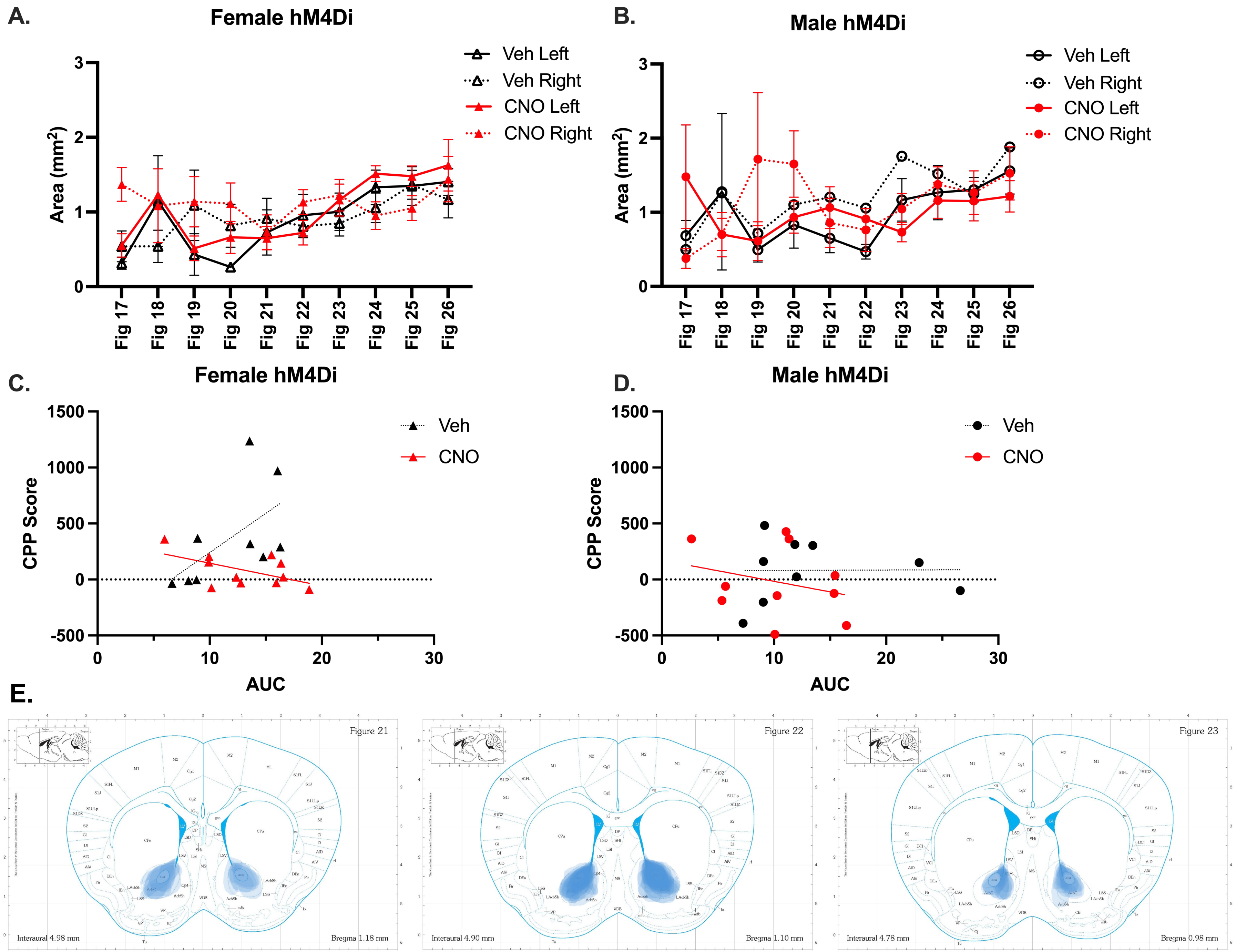

Supplemental Figure 8

References:

1. Gremel CM, Cunningham CL. Role of test activity in ethanol-induced disruption of place preference expression in mice. Psychopharmacology (Berl). 2007;191(2):195-202.
